# Proteomics reveals a therapeutic vulnerability via the combined blockade of APE1 and autophagy in lung cancer A549 cells

**DOI:** 10.1186/s12885-020-07111-w

**Published:** 2020-07-08

**Authors:** Shu-Ting Pan, Ji Zhou, Fang Yang, Shu-Feng Zhou, Tao Ren

**Affiliations:** 1 Department of Oral and Maxillofacial Surgery, The First Affiliated Hospital of Nanchang University, 17 Yongwai Main St, Nanchang, 330006 Jiangxi China; 2 Health Management Centre, The First Affiliated Hospital, Chengdu Medical College, 278 Baoguang St, Xindu Distr, Chengdu, 610500 Sichuan China; 3 Department of Pharmaceutical Sciences, College of Pharmacy, University of South Florida, 12901 Bruce B. Downs Boulevard, Tampa, Florida 33612 USA; 4 Oncology Department, The First Affiliated Hospital, Chengdu Medical College, 278 Baoguang St, Xindu Distr, Chengdu, 610500 Sichuan China

**Keywords:** Cisplatin, APE1, Autophagy, Chemotherapy, Non-small cell lung cancer, Apoptosis

## Abstract

**Background:**

Drug resistance is a major cause of therapeutic failure that is often associated with elevated autophagy and apurinic/apyrimidinic endonuclease 1 (APE1) expression. Herein, we investigated the role of APE1 and autophagy in A549 cells treated with cisplatin.

**Methods:**

SILAC proteomics was applied to obtain a panoramic view of cisplatin treatment in KRAS^G12S^-mutant A549 cells. Quantity analysis of cellular apoptosis and autophagy was based on flow cytometry. Western blotting was used to examine the expression levels of apoptosis- and autophagy-related proteins, as well as those of APE1. Knockdown of APE1 was achieved by RNA interference. Immunoprecipitation was further employed to reveal the molecular interaction of APE1, p53, and LC3 when A549 cells were exposed to cisplatin.

**Results:**

SILAC proteomics revealed that 72 canonical pathways, including base excision repair (BER) and autophagy signalling pathways, were regulated after cisplatin treatment in A549 cells. Cisplatin markedly induced autophagy and apoptosis in A549 cells, accompanied by remarkable APE1 increase. Suppression of autophagy enhanced the inhibition effect of cisplatin on cell growth, proliferation, and colony formation; however, APE1 inhibition enhanced the expression of LC3-I/II, suggesting that APE1 and autophagy are compensatory for cell survival to evade the anticancer action of cisplatin. Immunoprecipitation results revealed the triple complex of APE1-p53-LC3 in response to cisplatin plus CQ in A549 cells. Dual inhibition of APE1 and autophagy significantly enhanced cisplatin-induced apoptosis, which eventually overcame drug resistance in cisplatin-resistant A549 cells.

**Conclusions:**

Dual inhibition of APE1 and autophagy greatly enhances apoptosis in parental KRAS^G12S^-mutant A549 cells and cisplatin-resistant A549 cells via regulation of APE1-p53-LC3 complex assembly, providing therapeutic vulnerability to overcome cisplatin resistance in the context of KRAS^G12S^-mutant lung cancer.

## Background

Lung cancer is the leading cause of cancer-related death and remains a major clinical challenge with increasing incidence and mortality [1, 2]. Due to drug resistance, recurrence, and metastasis, the treatment efficacy of lung cancer remains unsatisfactory. A better understanding of the aetiology, pathogenesis, and molecular targets is required to develop novel therapeutic modalities. Somatic gene mutations, including *KRAS*, *EGFR*, and *TP53* mutations, is a major driver of lung cancer initiation [3]. Accumulating evidence has shown that not all gene mutations occur equally. In particular, compelling evidence suggests that RAS mutants function in an allele-specific manner, justifying the acquirement of a RAS allele-specific approach for RAS-driven cancer therapy [4–6]. Given the feature of allele specificity and the pivotal role of RAS in cellular events, including cell growth, cell survival, cell senescence, and cell death, novel strategies in a RAS allele-dependent manner are still required.

Autophagy is a cell survival-promoting mechanism following harsh stimuli and has been deeply implicated in cancer development and therapy [7–9]. Recently, targeting autophagy has been in the spotlight for cancer therapy via pharmacological inhibition alone or combination with other therapeutics [10, 11], providing insight into lung cancer therapy development. Cisplatin is one of the most frequently administered chemotherapeutic drugs for many solid tumours, including lung cancer. Mechanically, cisplatin kills cancer cells via interference with DNA synthesis and repair, subsequently inducing cell apoptosis [12]. However, there is limited clinical efficacy for cisplatin-based therapy because of drug resistance [13]. Several key factors contribute to cisplatin resistance, including autophagy [14] and apurinic/apyrimidinic endonuclease 1 (APE1) [15]. APE1 is a multifunctional protein with two major activities, DNA repair and transcriptional regulation [16]. Importantly, APE1 is often overexpressed in many tumours, contributing to disease progression, chemo-resistance and a poor prognosis [15, 17–20]. Our previous study found that APE1 is highly expressed in non-small cell lung cancer (NSCLC). Moreover, APE1 is a prognostic risk factor indicated by a poor overall survival [15, 19]. Herein, targeting APE1 might represent a therapeutic vulnerability for lung cancer, particularly, cisplatin-resistant lung cancer.

Thus, based on the aforementioned details, we hypothesized that APE1 and autophagy may contribute to lung cancer progression and drug resistance and that combined blockade of APE1 and autophagy enhances the therapeutic effect of cisplatin and overcomes cisplatin resistance in lung cancer. In the present study, we applied quantitative proteomics to identify the proteomic responses to cisplatin treatment in KRAS^G12S^-mutant A549 cells. Both APE1 and autophagy were involved in the cellular responses to cisplatin exposure. In A549 cells and cisplatin-resistant A549 cells, cisplatin-induced apoptosis was significantly enhanced via the combination of autophagy inhibition by chloroquine (CQ) and APE1 knockdown by siRNA with the involvement of p53 activation.

## Methods

### Chemicals and reagents

CDDP was purchased from Selleckchem Inc. (Houston, TX, USA). ^13^C_6_-L-lysine, L-lysine, ^13^C_6_^15^N_4_-L-arginine, L-arginine, Dulbecco’s modified Eagle’s medium (DMEM)/F12 for SILAC, APE1 siRNA, dimethyl sulfoxide (DMSO), 2-(4,5-dimethylthiazol-2-yl)-2,5-diphenyltetrazolium bromide (MTT), bovine serum albumin, and Dulbecco’s phosphate-buffered saline (PBS) were obtained from Sigma-Aldrich (St. Louis, MO, USA). 6-Diamidino-2-phenylindole (DAPI), Opti-minimal Essential Medium (MEM), Lipofectamine 2000, and the negative control siRNA were purchased from Invitrogen Inc. (Carlsbad, CA, USA). The Annexin V-phycoerythrin (PE) apoptosis detection kit was purchased from BD Biosciences Inc. (San Jose, CA, USA). The Cyto-ID® Autophagy detection kit was obtained from Enzo Life Sciences Inc. (Farmingdale, NY, USA). The Western blotting substrate, Pierce™ bicinchoninic acid (BCA) protein assay kit, skim milk, and radioimmunoprecipitation assay buffer (RIPA) were purchased from Thermo Fisher Scientific Inc. (Hudson, NH, USA). The polyvinylidene difluoride (PVDF) membrane was obtained from Bio-Rad Inc. (Hercules, CA, USA). The antibody against human β-actin was obtained from Santa Cruz Biotechnology Inc. (Dallas, TX, USA). The remaining primary antibodies for signalling proteins related to apoptosis and autophagy were purchased from Cell Signaling Technology Inc. (Beverly, MA, USA).

### Cell line and cell culture

The human lung cancer cell line A549 (KRAS^G12S^) was obtained from Chinese Academy of Science Cellbank (Shanghai, China) and was cultured in RPMI1640 medium supplemented with 10% heat-inactivated foetal bovine serum (FBS). The cells were maintained at 37 °C in a 5% CO_2_/95% air humidified incubator.

### Cell viability determination

The MTT assay was used to evaluate cell viability. Briefly, cells were seeded in 96-well plates at a density of 7.0 × 10^3^ cells/well. After 24 h. of incubation, the cells were treated for 48 h. The absorbance was measured using a Synergy™H4 Hybrid microplate reader (BioTek, Winooski, VT, USA) at wavelengths of 560 nm (MTT formazan) and 670 nm (background).

### Quantitative proteomics

Quantitative proteomic experiments were performed using a stable isotope labelling by amino acids in cell culture (SILAC)-based approach to identify the molecular targets of CDDP in the treatment of A549 cells as previously described [21]. Briefly, A549 cells were cultured in DMEM/F12 medium (for SILAC) with (heavy) or without (light) stable isotope-labelled amino acids (^13^C_6_ L-lysine and ^13^C_6_^15^N_4_ L-arginine) and 10% dialyzed FBS. After treatment with CDDP (5 μM) for 24 h., the cell samples were harvested, lysed, and quantified. Next, an equal amount of heavy and light protein samples were combined to reach a total volume of 50 μL containing 400 μg of protein, and the combined protein sample was digested and desalted. Next, the peptide mixtures (5 μL) were subjected to the hybrid linear ion trap. The peptide SILAC ratio was calculated using MaxQuant version 1.2.0.13. The proteins were identified using Scaffold 4.3.2, and the pathway was analysed using ingenuity pathway analysis (IPA) from QIAGEN Inc.

### Quantification of cellular apoptosis

Cell apoptosis was evaluated using the Annexin V-PE apoptosis detection kit as previously described [21]. Briefly, the cells were collected after treatment and resuspended in 1× binding buffer with 5 μL of Annexin V-PE and 5 μL of 7-amino-actinomycin D (7-AAD) at 1 × 10^5^ cells/mL in a total volume of 150 μL. The cells were gently mixed and incubated in the dark for 15 min at room temperature. The binding buffer (100 μL) was then added to each tube, and the number of apoptotic cells was quantified using flow cytometry and collecting 10,000 events for analysis.

### Quantification of cellular autophagy

Cell autophagy was examined using flow cytometry as previously described [21]. Briefly, the cells were collected after treatment and resuspended in 250 μL of assay buffer containing 5% FBS, and Cyto-ID® Green stain solution (250 μL) was added to each tube and mixed gently. After 20 min of incubation at room temperature in the dark, the cells were collected by centrifugation, washed once and analysed using the green (FL1) channel of flow cytometry.

### Confocal fluorescence microscopy

Confocal microscopy was performed to evaluate the cellular autophagy level in A549 cells after treatment with 5 μM CDDP, 10 μM CQ, and 5 μM CDDP + 10 μM CQ using the Cyto-ID autophagy detection kits as previously described [21]. The fluorescence was assessed using TCS SP2 laser scanning confocal microscopy (LSCM).

### Western blotting assay

The protein expression level was examined using Western blotting. Protein samples were extracted using RIPA buffer, the protein concentrations were measured using the BCA kit, and an equal amount of protein was separated by SDS-PAGE. The corresponding primary and secondary antibodies were applied to evaluate the expression levels of targeted proteins. Visualization was performed using the Bio-Rad ChemiDoc™ XRS system, and the blot bands were analysed using Image Lab 3.0.

### RNA interference

Small interfering RNA-mediated gene silencing was performed to investigate the role of APE1 in cisplatin-induced apoptosis and autophagy in A549 cells according to the manufacturer’s instructions. A549 cells were transfected with the negative control siRNA and APE1-siRNA using Lipofectamine 2000. The protein samples were collected and kept at − 80 °C for further analysis.

### Immunoprecipitation

The interaction between APE1 and p53 was examined using immunoprecipitation as previously described [22]. After 24 h. of treatment, A549 cells were lysed in pre-chilled cell lysis buffer [50 mM Tris-HCl (pH 7.4), 150 mM NaCl, 1 mM EDTA, 1% NP40, protease inhibitors] for 5 min. The lysates were precleared with 20 μL of Proteins A/G (Invitrogen; Thermo Fisher Scientific, Inc.) at 4 °C for 45 min, followed by incubation with APE1 or p53 antibody overnight at 4 °C. Following immunoprecipitation, the samples were incubated with protein G for 3 h. at 4 °C. Thereafter, the samples were washed with lysis buffer five times to remove any un-precipitated proteins before boiling in SDS buffer for 5 min. The elution was analysed for precipitated APE1 or p53 protein using Western blotting analysis. Normal rabbit IgG antibody was used as a negative control. The antibodies used were as follows: APE1 (1:500), p53 (1:500), and normal rabbit IgG (1:1000).

### Statistical analysis

The data were expressed as means ± standard deviation (SD). One-way analysis of variance (ANOVA) followed by Tukey’s multiple comparison procedure was used for comparisons of multiple groups. The value of *P*<0.05 was considered statistically significant. The assays were performed at least three times independently.

## Results

### Overview of the proteomic response to cisplatin treatment in A549 cells

Until now, a lack of effective therapeutics persists for *KRAS* mutation-driven lung cancer. Compelling evidence has shown that RAS mutations vary and has spurred the development of new therapeutic vulnerabilities in a RAS allele-specific manner. To explore possible therapeutic targets, we applied SILAC-based proteomics to reveal the full spectrum of the molecular interactome in A549 cells in the context of the KRAS^G12S^ mutant following cisplatin exposure. We evaluated the proteomic responses to cisplatin (5 μM) treatment and identified at least 3262 protein molecules responding to cisplatin treatment, including APE1, p53, LC3-I/II, and many other functional proteins involved in DNA damage repair, cell proliferation, cell cycle, cellular metabolism, apoptosis, and autophagy. Subsequent IPA analysis revealed 1013 cellular functional proteins (450 proteins were upregulated; 563 proteins were downregulated) and 72 canonical signalling pathways that are involved in cell cycle control of chromosomal replication, RNA signalling, the BER pathway, DNA double-strand break repair by non-homologous end joining, ILK signalling, mismatch repair, mTOR signalling, ATM signalling, EGF signalling, telomere extension by telomerase, the spliceosomal cycle, the role of CHK protein in cell cycle checkpoint control, glycolysis I, gluconeogenesis I, DNA methylation and transcriptional repression signalling, the NRF2-mediated oxidative stress response, apoptosis, and autophagy (see Additional file 2: Supplementary Fig. 32A-B). As shown in Supplementary Fig. 32C, among the proteins in the BER pathway, APE1 expression was increased. Moreover, autophagy participated in the cellular responses to cisplatin treatment in A549 cells, as evident from the alteration in the expression of MAP1LC3 (also named as LC3) after cisplatin treatment (see Additional file 2: Supplementary Fig. 32D). Collectively, we speculated that both BER and autophagy pathways are involved in cisplatin-stimulated cellular responses in KRAS^G12S^-mutant A549 cells. Thus, we subsequently investigated their roles in responses to cisplatin treatment.

### Cisplatin induces autophagy and apoptosis and increases APE1 expression

**Fig. 1:**
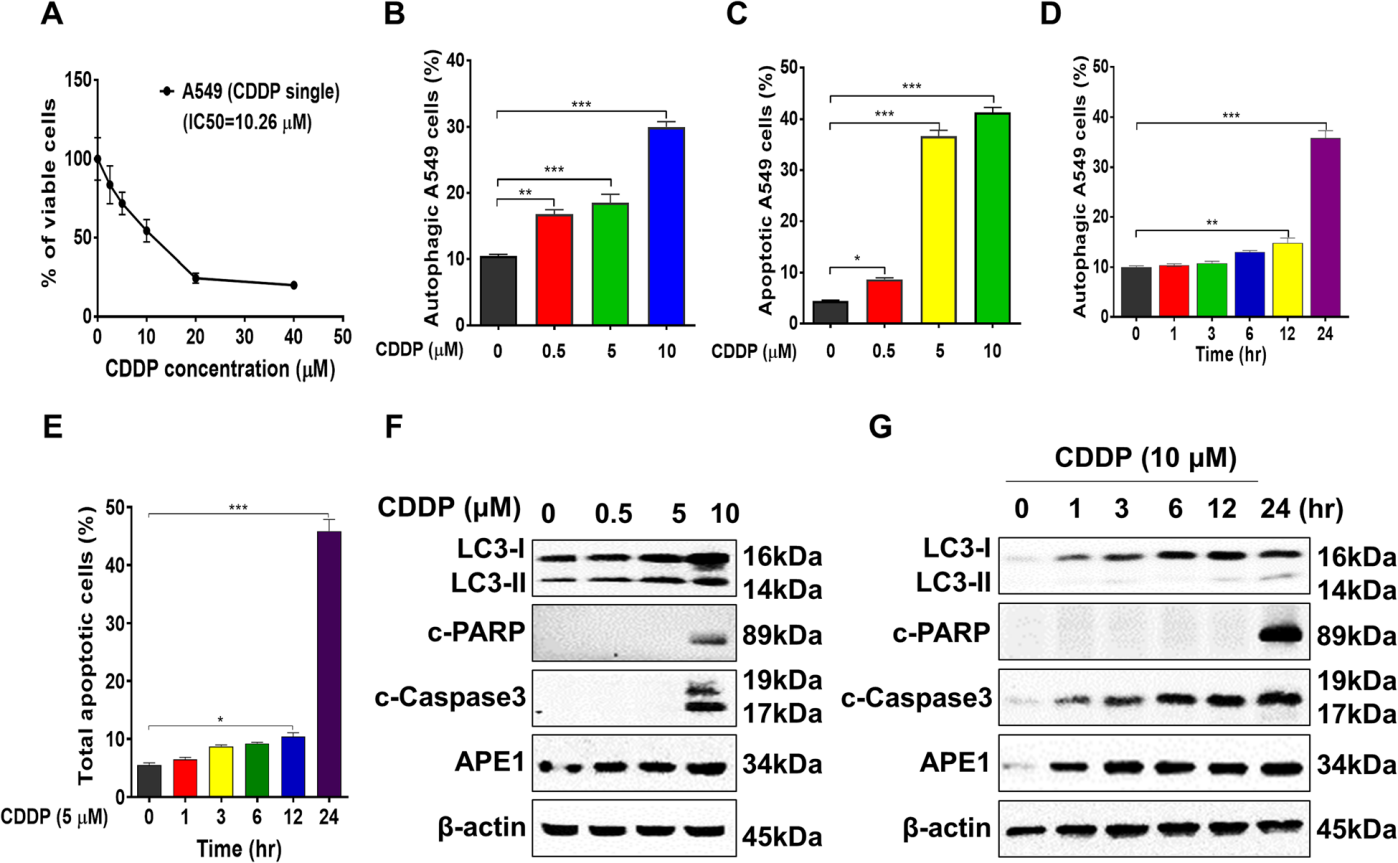
Cisplatin induces autophagy and apoptosis. **a** Cisplatin markedly inhibited A549 cell proliferation in a dose-dependent fashion. The IC_50_ value was 10.26 μM. **b** A549 cells were treated with cisplatin at 0.5, 5, and 10 μM for 24 h. Flow cytometry was used to determine cisplatin-induced autophagy. **c** A549 cells were treated with cisplatin at 0.5, 5, and 10 μM for 24 h. Flow cytometry was used to determine cisplatin-induced apoptosis. **d** Cisplatin-induced autophagy was performed in a time-dependent manner. A549 cells were treated with cisplatin at 5 μM for 1, 3, 6, 12 and 24 h. **e** A549 cells were treated with 5 μM cisplatin for 1, 3, 6, 12, and 24 h. Flow cytometry was used to determine apoptosis. **f** The expression levels of apoptosis- and autophagy-related proteins, as well as those of APE1, were examined using Western blotting after treatment with 0.5, 5, and 10 μM cisplatin for 24 h (see Additional file 1: Supplementary Figure 1-3). **g** The expression levels of apoptosis- and autophagy-related proteins, as well as APE1, were examined using Western blotting after treatment with 5 μM cisplatin for 1, 3, 6, 12, and 24 h (see Additional file 1: Supplementary Figure 4-7).

As observed above regarding the proteomic responses to cisplatin treatment, autophagy and apoptosis were involved. Thus, we tested cisplatin-induced autophagy and apoptosis in A549 cells. Cisplatin decreased the number of viable A549 cells concentration-dependent manner (Fig. 1a). Cisplatin was reported to induce autophagy and apoptosis in various cancers, including lung cancer [23–25], and autophagy is related to chemo-resistance, providing a cell-protective mechanism that can promote tumour cell survival following different stresses, including chemotherapeutic treatment [26]. We showed that cisplatin treatment led to concentration- and time-dependent increases in autophagy in KRAS^G12S^-mutant A549 cells and markedly increased the expression of LC3-I/II (Fig. 1b, d, f and g). Accompanying the autophagy phenomenon, the dose and timescale experiments showed that cisplatin treatment also induced remarkable apoptosis in A549 cells (Fig. 1c and e). Activation by the cleavage of PARP and caspase 3 only occurred at a high concentration of cisplatin (10 μM; Fig. 1f-g). Notably, the proteomic study showed that APE1 is involved in the responses to cisplatin treatment, and we previously showed that high APE1 expression in patients with NSCLC was positively correlated with poor overall survival, implying that APE1 is a prognostic risk factor [15, 19]. In the dose and timescale experiments, the expression of APE1 was dramatically increased following cisplatin treatment in A549 cells (Fig. 1f-g). Together, the results showed that cisplatin-induced autophagy and APE1 expression could counteract the apoptotic effect in KRAS^G12S^-mutant A549 cells, suggesting the inhibition of autophagy or APE1 can enhance the cell-killing effect of cisplatin.

### Inhibition of autophagy enhances cisplatin-induced apoptosis

**Fig. 2:**
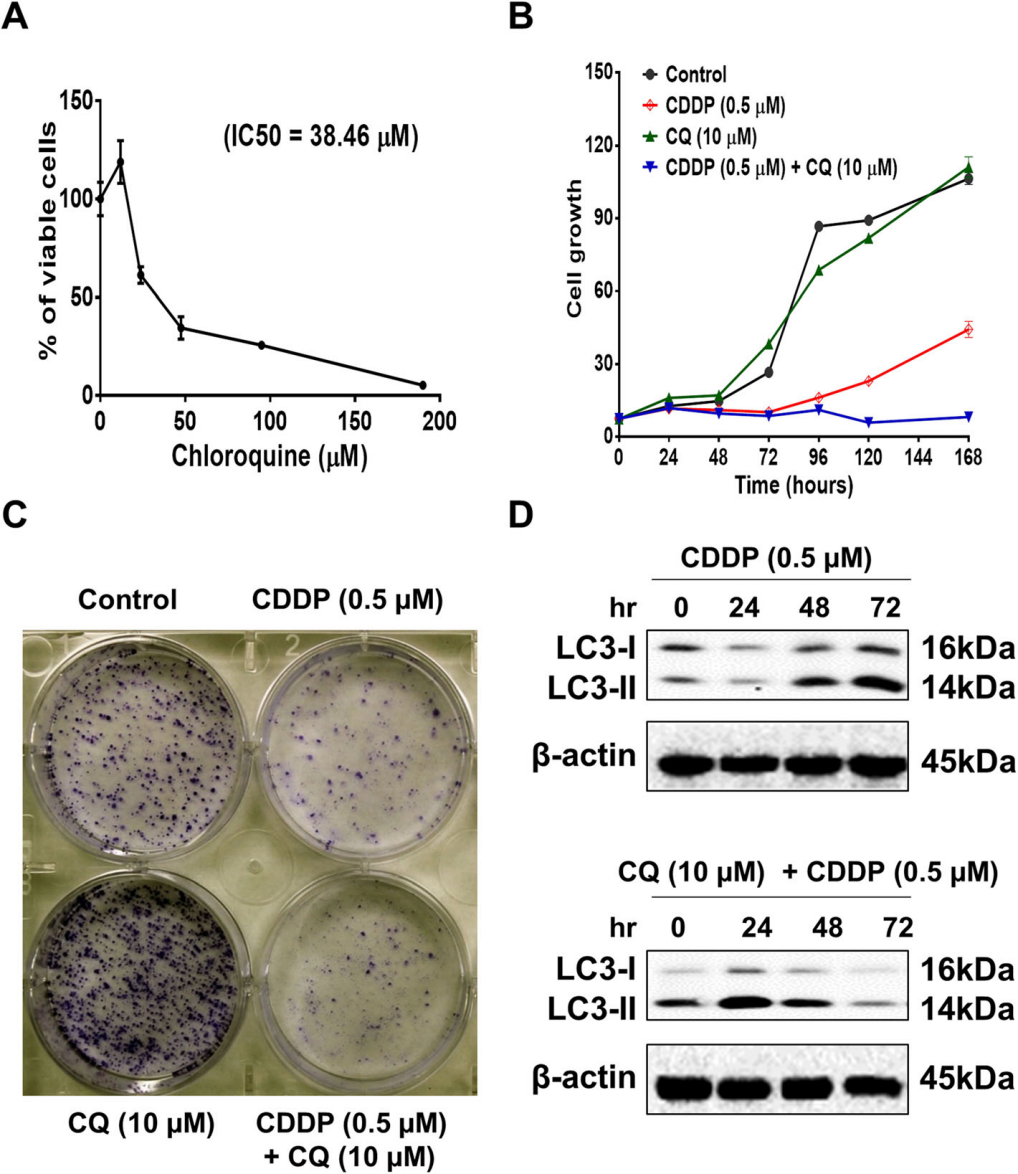
Inhibition of autophagy enhances the inhibitory effect of cisplatin in A549 cells. **a** Inhibition of autophagy by CQ markedly decreased A549 cell viability. The IC_50_ value was 38.46 μM. **b** CQ (10 μM) enhanced the inhibitory effect of 0.5 μM cisplatin on cell proliferation. **c** CQ enhanced the inhibitory effect of cisplatin on cell colony formation. **d** CQ suppressed the expression level of cisplatin-induced autophagy (see Additional file 1: Supplementary Figure 8-9).

**Fig. 3:**
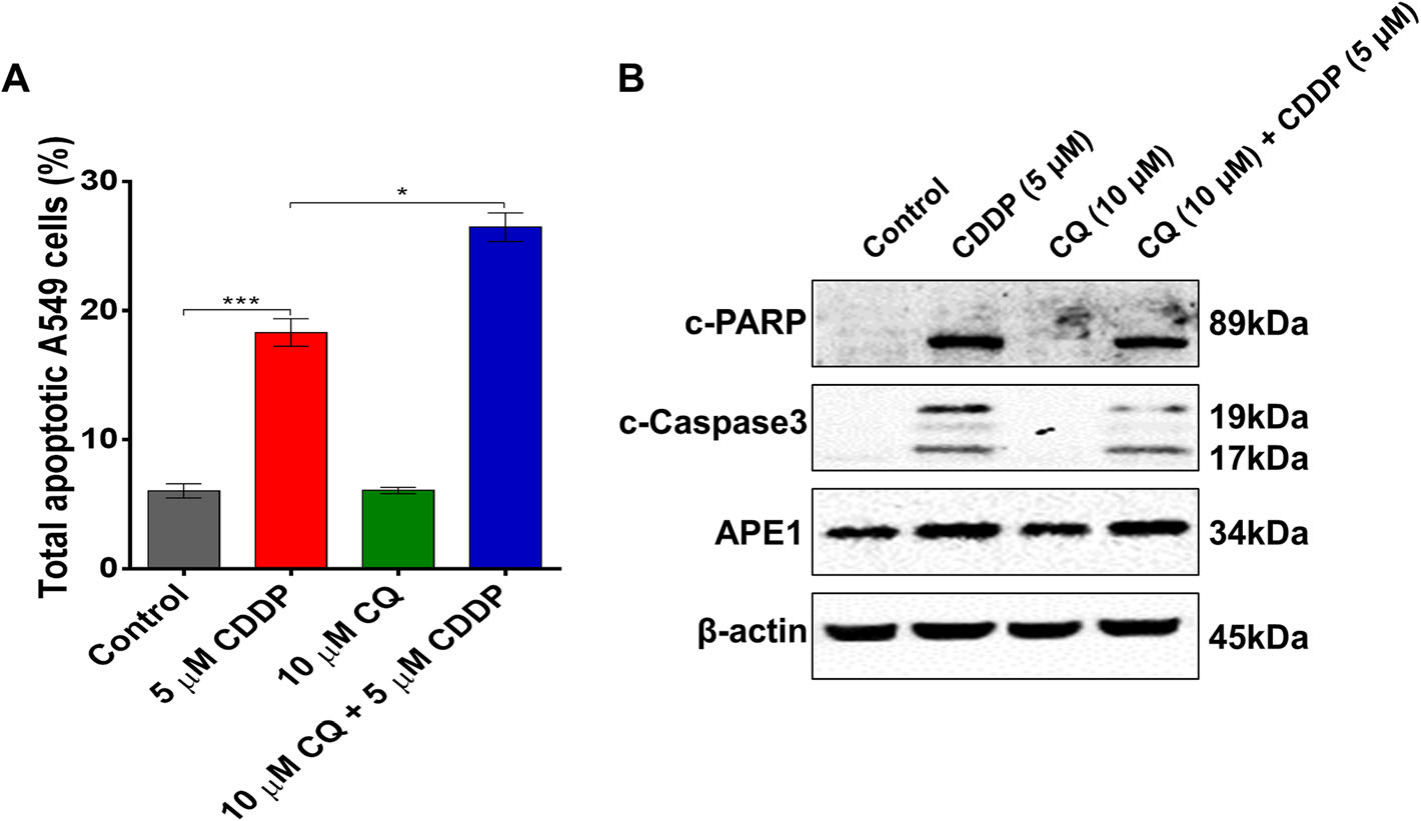
Inhibition of autophagy enhances cisplatin-induced apoptosis in A549 cells. **a** Inhibition of autophagy by CQ markedly enhanced cisplatin-induced apoptosis in A549 cells. **b** Western blotting showed that CQ enhanced cisplatin-induced apoptosis in A549 cells (see Additional file 1: Supplementary Figure 10-12).

Recently, compelling evidence has shown that the inhibition of autophagic flux effectively enhances the tumour-suppressive effect of MAPK signalling inhibitors in the treatment of cancer [10, 11]. Chloroquine (CQ), originally used as an anti-malarial drug, has been in the spotlight as an autophagy inhibitor and a novel chemotherapeutic agent [10, 27]. CQ can diffuse through cell membranes and accumulate in cellular lysosomes, repressing autophagosome fusion with lysosomes [28, 29]. Thus, we tested the effect of CQ on cell growth and colony formation alone or in combination with cisplatin at low concentration (0.5 μM) in A549 cells. First, we showed that CQ decreased A549 cell viability at a high dose with an IC_50_ value at 38.46 μM (Fig. 2a). Next, we tested the effect of CQ on cell growth and viability alone and in combination with cisplatin. Treatment of A549 cells with 10 μM CQ did not affect cell growth compared with the control; however, 0.5 μM cisplatin remarkably suppressed cell growth after 1 week (Fig. 2b). Notably, combinatorial treatment of cells with CQ and cisplatin inhibited cell growth (Fig. 2b). Furthermore, co-treatment of A549 cells with CQ and cisplatin showed a higher inhibitory effect on cell colony formation than CQ or cisplatin treatment alone (Fig. 2c). Similarly, treatment of A549 cells with cisplatin alone increased the expression of LC3-I/II, whereas combinatorial treatment of cells with CQ and cisplatin decreased the expression of LC3-I/II (Fig. 2d), suggesting that inhibition of autophagic flux enhances the effect of cisplatin. Indeed, co-treatment of A549 cells with CQ and cisplatin enhanced cisplatin-induced apoptosis, whereas no effect of CQ on A549 cell apoptosis was observed (Fig. 3a-b). Intriguingly, APE1 expression was increased with both cisplatin treatment alone and in combination with CQ (Fig. 3b), suggesting APE1 counteracts the effect of dual treatment of cisplatin and CQ. Taken together, these data showed that inhibition of autophagic flux enhances the cisplatin-induced cell growth-suppressive effect and apoptosis.

### Inhibition of APE1 stimulates autophagy in A549 cells

**Fig. 4:**
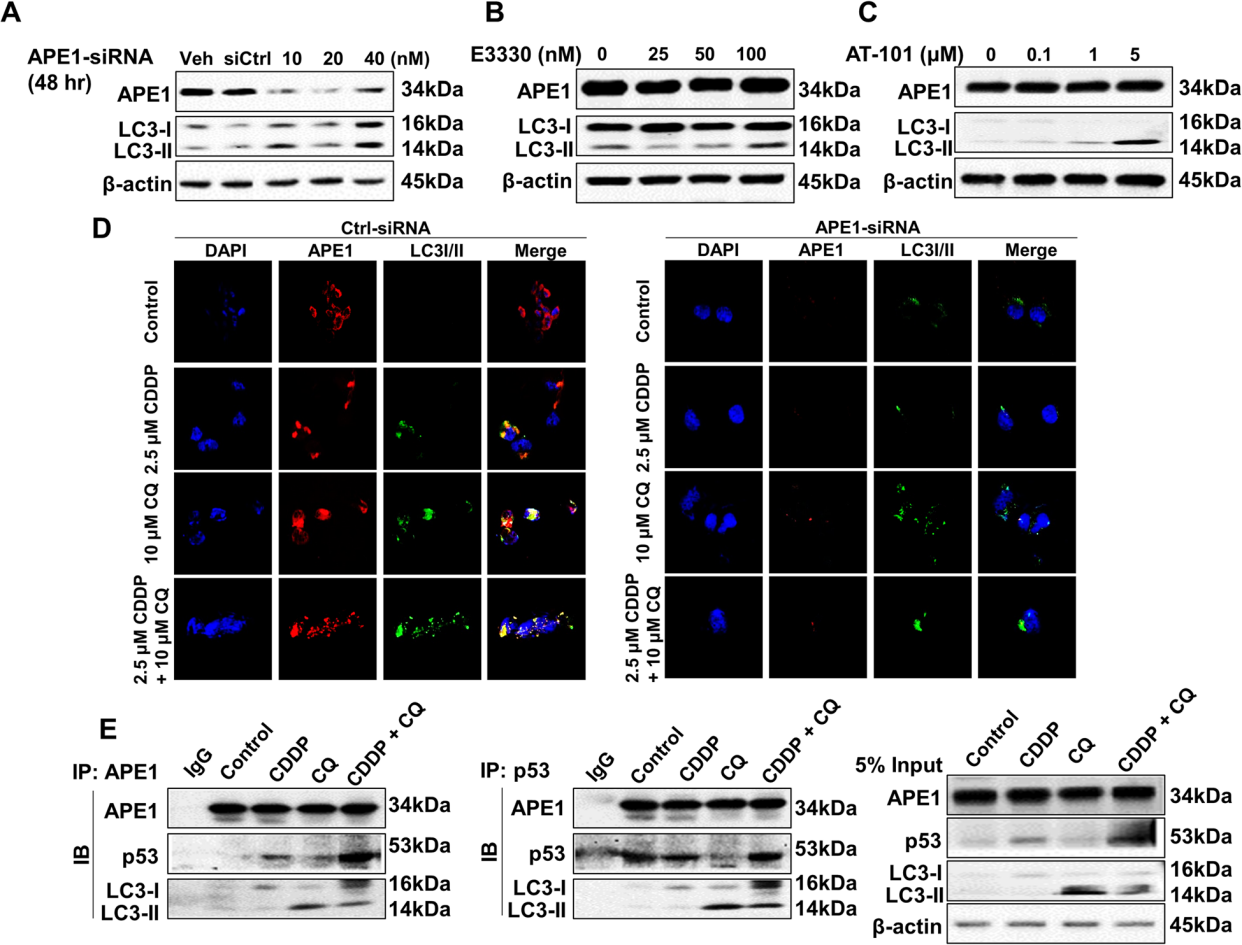
APE1 knockdown induces autophagy. **a** Si-RNA-mediated APE1 knockdown increased LC3-I/II expression (see Additional file 1: Supplementary Figure 13-14). **b** and **c** Inhibition of APE1 via E3330 or AT-101 increased LC3-I/II expression in A549 cells (see Additional file 1: Supplementary Figure 15-16). **d** Confocal microscopy showed that depletion of APE1 affected LC-3I/II localization. **e** Immunoprecipitation showed the interaction between APE1, LC3, and p53 in A549 cells (see Additional file 1: Supplementary Figure 17-20).

We first tested the effect of the mono-inhibition of APE1 on autophagy in A549 cells, which show high APE1 expression [30]. SiRNA-mediated knockdown or chemical inhibition of APE1 increased the expression of LC3-II (Fig. 4a-c). Confocal microscopic examination also showed that knockdown of APE1 increased autophagy compared with control siRNA (Fig. 4d). However, in the presence of cisplatin and CQ treatment alone or together, knockdown of APE1 not only reduced the expression level of LC3-I/II but also prevented nuclei accumulation (Fig. 4d). Additionally, the merged images showed co-localization of APE1 and LC3-I/II in A549 cells (Fig. 4d). Next, we performed immunoprecipitation to examine the possible interactions between LC3-I/II and key proteins involved in DNA damage, including APE1 and p53. Immunoprecipitation showed complex formation of APE1-p53-LC3 in response to cisplatin and CQ alone or in combination (Fig. 4e). Combinatorial treatment enhanced the formation of APE1-p53 but decreased LC3-II (Fig. 4E). Collectively, the data suggested that suppression of APE1 induces LC3-II expression, and autophagy plays an important role in cell survival in response to APE1 deficiency with the involvement of the interaction between APE1, p53, and LC3.

### Combined blockade of APE1 and autophagy promotes cisplatin-induced apoptosis in A549 cells

**Fig. 5:**
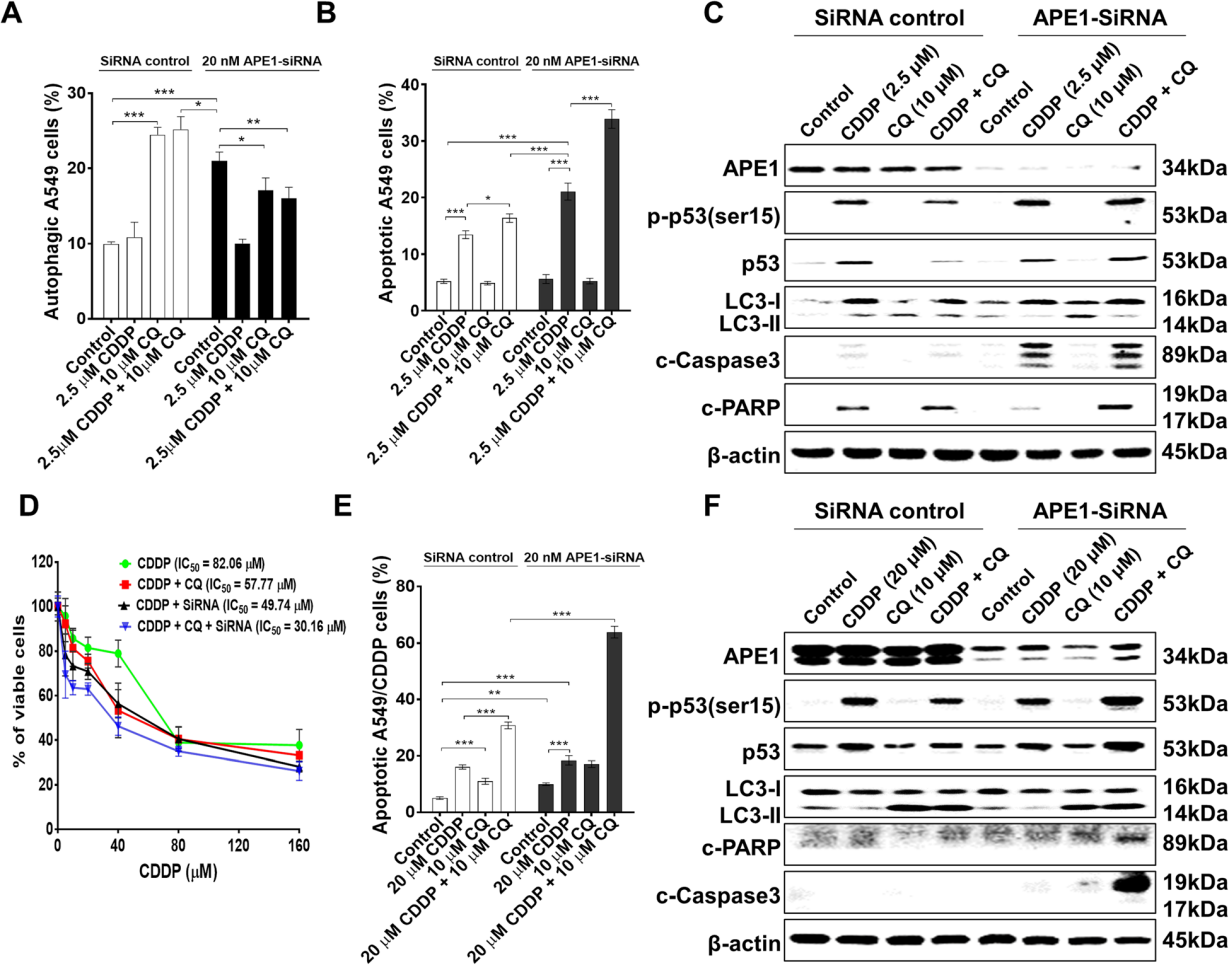
Combined blockade of autophagy and APE1 increases chemosensitivity and overcomes cisplatin resistance. **a** and **b** Knockdown of APE1 enhanced cisplatin-induced apoptosis in combination with autophagy inhibition by CQ. **c** Western blotting showed the enhancing effect of APE1 silencing on cisplatin-induced apoptosis in combination with autophagy inhibition by CQ (see Additional file 1: Supplementary Figure 21-26). **d** Knockdown of APE1 enhanced cisplatin sensitivity in combination with autophagy inhibition in cisplatin-resistant A549 cells. **e** Knockdown of APE1 enhanced cisplatin-induced apoptosis in combination with autophagy inhibition in cisplatin-resistant A549 cells. **f** Western blotting showed the enhancing effect of APE1 silencing on cisplatin-induced apoptosis in combination with autophagy inhibition in cisplatin-resistant A549 cells (see Additional file 1: Supplementary Figure 27-31).

As demonstrated above, inhibition of autophagy or APE1 alone did not show an effective cell-killing effect in A549 cells because of the compensatory effect of autophagy and APE1. Thus, we speculated that combined blockade of autophagy and APE1 would further enhance the cell-killing effect of cisplatin in A549 cells. We applied a low concentration of cisplatin (2.5 μM), but the results showed no effect on autophagy in A549 cells (Fig. 5a). Knockdown of APE1 attenuated autophagy in the presence of cisplatin or CQ treatment alone or in combination; however, the depletion of APE1 enhanced apoptosis following treatment with cisplatin alone or in combination with CQ. Notably, the combinatorial treatment of CQ and cisplatin exerted the most effective apoptotic effect in the presence of APE1 knockdown (Fig. 5b). The protein expression level of LC3 and activation by the cleavage of PARP and caspase 3 reflected the autophagy and apoptosis (Fig. 5c). Notably, emerging data found that cisplatin stimulates p53 activity [31]. Consistent with the results, our data showed that cisplatin treatment alone or in combination with CQ increased p53 expression, as well as p-p53 (ser15) expression (Fig. 5c), suggesting that p53 is involved in the response to cisplatin treatment in A549 cells. Taken together, these results suggest that combined blockade of APE1 and autophagy enhances cisplatin-induced apoptosis with the involvement of p53 activation in A549 cells.

### Dual repression of APE1 and autophagy reverses cisplatin resistance in cisplatin-resistant A549 cells

Acquired cisplatin resistance is the major cause of chemotherapy failure in the treatment of lung cancer; thus, we speculated that dual inhibition of APE1 and autophagic flux would overcome cisplatin resistance. We tested the effects on cell proliferation and apoptosis in acquired cisplatin-resistant A549 cells (parental A549 cells were exposed to cisplatin to develop the acquired resistant cell line, named A549/CDDP [32]) following dual inhibition of both the APE1 and autophagy. The cell viability was decreased in A549/CDDP cells receiving combinatorial treatment with cisplatin and CQ or cisplatin and APE1 siRNA compared with the vehicle or mono-treatment (Fig. 5d). Moreover, the most inhibitory effect on cell viability was observed in A549/CDDP cells receiving the combinatorial treatment with cisplatin, CQ, and APE1 knockdown (Fig. 5d). Next, the A549/CDDP cells were treated with a high concentration of cisplatin (20 μM) alone or in combination. Co-treatment with cisplatin and CQ resulted in increased apoptosis compared with mono-treatment in A549/CDDP cells, but co-treatment with APE1-SiRNA and cisplatin or CQ showed no enhancement in apoptosis compared with mono-treatment (Fig. 5e). The most potent apoptotic effect was observed in A549/CDDP cells treated with cisplatin, CQ, and APE1-SiRNA together. Knockdown of APE1 markedly sensitized A549/CDDP cells to the cisplatin/CQ combinatorial treatment (Fig. 5e). Protein expression also showed that the activation of p53 and PARP and caspase 3 cleavage occurred in A549/CDDP cells treated with cisplatin, CQ, and APE1-SiRNA together (Fig. 5f). No significant cleavage of PARP and caspase 3 was observed in the absence of APE1-SiRNA (Fig. 5F), suggesting the enhancing role of APE1 knockdown in apoptosis in cisplatin-resistant cells exposed to an autophagy inhibitor. Together, the data suggest that the combined blockade of APE1 and autophagy may be an effective strategy to overcome cisplatin resistance.

## Discussion

Lung cancer is the leading cause of cancer death, and a lack of efficacious therapeutics exists. Cisplatin, the most important chemotherapeutic drug in lung cancer therapy, has shown limited clinical efficacy due to drug resistance. Autophagy and other key cellular events, including the DNA damage repair response, are involved in chemo-drug resistance. Notably, there is increasing attention on the genetic context dependence in lung cancer therapy. Compelling evidence has shown that oncogenic RAS mutations vary, although they all promote cancer cell proliferation [4–6]. Zhong et al. found that inhibition of RAS-AKT-mTOR signalling and blockage of late stage autophagy could synergistically enhance the cytotoxicity of a tumour suppressor gene ARHI [33]. Specific RAS alleles exhibit differential biochemical features, displaying preferential signalling output and favouring differential downstream effectors that are subject to differential feedforward and feedback regulations. Therefore, individualized therapeutics are advocated in cancer therapy.

In this study, we first applied SILAC proteomics to obtain a panoramic view of cisplatin treatment in KRAS^G12S^-mutant A549 cells. At least 3262 protein molecules responded to cisplatin treatment and included APE1, p53, and LC3-I/II, which are involved in DNA damage repair, cell proliferation, apoptosis, and autophagy. Subsequent IPA analysis revealed 72 canonical signalling pathways including the BER pathway, DNA double-strand break repair, and autophagy pathways. Autophagy is a well-known cell-protective mechanism related to tumor progression, drug-resistance, and survive [8], and blockade of RAS/RAF/MEK/ERK signalling flux promotes autophagy [10], suggesting that inhibition of autophagy is beneficial. Kinsey et al. [10] and Bryant et al. [11] have shown synergistic antitumor effects of autophagy inhibition and MAPK inhibition in RAS-driven cancers, including pancreatic ductal adenocarcinoma, melanoma, and colorectal cancer, in preclinical settings [10, 11]. This combined blockade of autophagy with other therapeutics revealed a novel therapeutic vulnerability to treat RAS-driven cancers, including lung cancer. Based on our previous research concerning BER pathway in platinum-resistance of lung cancer [30] and present proteomic results, we herein aimed to investigate whether BER and autophagy have interaction upon cisplatin treatment in lung cancer cells. By methods of flow cytometry, fluorescence microscopy, Western blotting and RNA interference, we found that cisplatin markedly induced autophagy and apoptosis in A549 cells, accompanied by remarkable increase of DNA repair protein APE1. Suppression of autophagy enhanced the inhibition effect of cisplatin on cell growth, proliferation, and colony formation. The combination treatment of CQ, an autophagy inhibitor, with cisplatin dramatically enhanced cisplatin-induced apoptosis.

Moreover, APE1 is a major contributor to cisplatin resistance in lung cancer [15]. In the present study, knockdown of APE1 enhanced cisplatin-induced apoptosis in both A549 cells and cisplatin-resistant A549 cells. Noteworthy, APE1 knockdown significantly synergized the apoptosis-inducing effect of cisplatin plus CQ. This dual inhibition of APE1 and autophagy could minimize the curative concentration of cisplatin in cisplatin-resistant A549 cells. The lower concentration of cisplatin was beneficial in reducing the side effects of chemotherapy that commonly occur in clinical settings. Besides, the specific targeting of autophagy without affecting other cellular processes has drawn a great attention of researchers. Mutations in the RAS pathway are often associated with the high levels of autophagy that are required to maintain cancer cell metabolism [34, 35]. The optimal dosage of autophagy inhibitors and timing of inhibition are vital parameters for maximal therapeutic efficiency. Hopefully, Levy et al. reported that the treatment of CQ as an autophagy inhibitor in some cancer patients showed no adverse toxicity for extended time periods [36]. This demonstrates that long-term treatment with lysosomal autophagy inhibitors is feasible. Provided that cancer cells are more dependent than normal tissues on autophagy, even a drug that causes some normal tissue toxicity can have a valuable therapeutic window for an effective cancer treatment [8]. In inducible Atg7-knockout mice, the growth of KRAS-driven lung tumors was significantly inhibited before any signs of neurotoxicity [37], indicating that therapeutic window for autophagy inhibition exists in some cancers.

Additionally, our previous data found that promoting p53 intracellular stability by interfering with APE1 is a possible mechanism in genistein-induced apoptosis [38]. In the present study, we applied immunoprecipitation to explore the possible interactions between LC3-I/II and key proteins involved in DNA damage, including APE1 and p53. An interesting triple complex comprising APE1-p53-LC3 was formed in response to cisplatin plus CQ in A549 cells. Taken together, our results suggest that dual inhibition of APE1 and autophagy could enhance chemo-sensitivity and overcome cisplatin resistance by boosting apoptosis via the modulation of APE1-p53-LC3 complex assembly in a KRAS^G12S^ context. Whether the current findings regarding the cellular events recapitulate other *RAS* mutations in lung cancer or *KRAS*^*G12S*^ mutation in other cancer types in response to cisplatin treatment is unknown and warrants further investigation to better tailor specific therapeutic vulnerabilities for lung cancer treatment [3].

## Conclusions

In summary, this study revealed a proteomic response to cisplatin in KRAS^G12S^-mutant A549 cells. APE1, p53, and LC3-I/II were identified to be involved in DNA damage repair, cell proliferation, apoptosis, and autophagy. Dual inhibition of APE1 and autophagy synergistically enhanced cisplatin-induced apoptosis via the regulation of APE1-p53-LC3 complex assembly. This novel combination strategy is of great potential to overcome cisplatin resistance in the context of KRAS^G12S^-mutant lung cancer.

## Supplementary information

**Additional file 1: Figure S1-S31.** Original gels and blot images. Image Lab 3.0 software (Bio-Rad, USA) was used to analyse the blots. The cropping of the blots was labelled with the symbol of “↓”. Corresponding uncropped full-length blots are presented in Supplementary Figure [1–31].

**Additional file 2: Figure 32.** SILAC-based proteomics identifies cellular response molecules and some related signalling pathways in cells. **A** Twenty-seven cranial signalling pathways. **B** Hot-point picture. **C** BER signalling pathway. **D** Autophagy signalling.

## Data Availability

The datasets used in the current study are available from the corresponding author on reasonable request.

## Abbreviations

APE1: Apurinic/apyrimidinic endonuclease 1
NSCLC: Non-small cell lung cancer
CDDP: Cisplatin
CQ: Chloroquine
DMSO: Dimethyl sulfoxide
DMEM: Dulbecco’s modified Eagle’s medium
PBS: Dulbecco’s phosphate-buffered saline
FBS: Foetal bovine serum
SILAC: Stable isotope labelling by amino acids in cell culture
LSCM: Laser scanning confocal microscope
DAPI: 6-diamidino-2-phenylindole
MTT: 2-(4,5-dimethylthiazol-2-yl)-2,5- diphenyltetrazolium bromide
RIPA: Radioimmunoprecipitation assay buffer
PVDF: Polyvinylidene difluoride
IPA: INGENUITY Pathway Analysis
