# Supplementary material for: Proteomics reveals a therapeutic vulnerability via the combined blockade of APE1 and autophagy in lung cancer A549 cells
Source: BMC Cancer. 2020 Jul 8;20:634. doi: 10.1186/s12885-020-07111-w (PMC7346405; doi:10.1186/s12885-020-07111-w)

Supplementary Figure 1

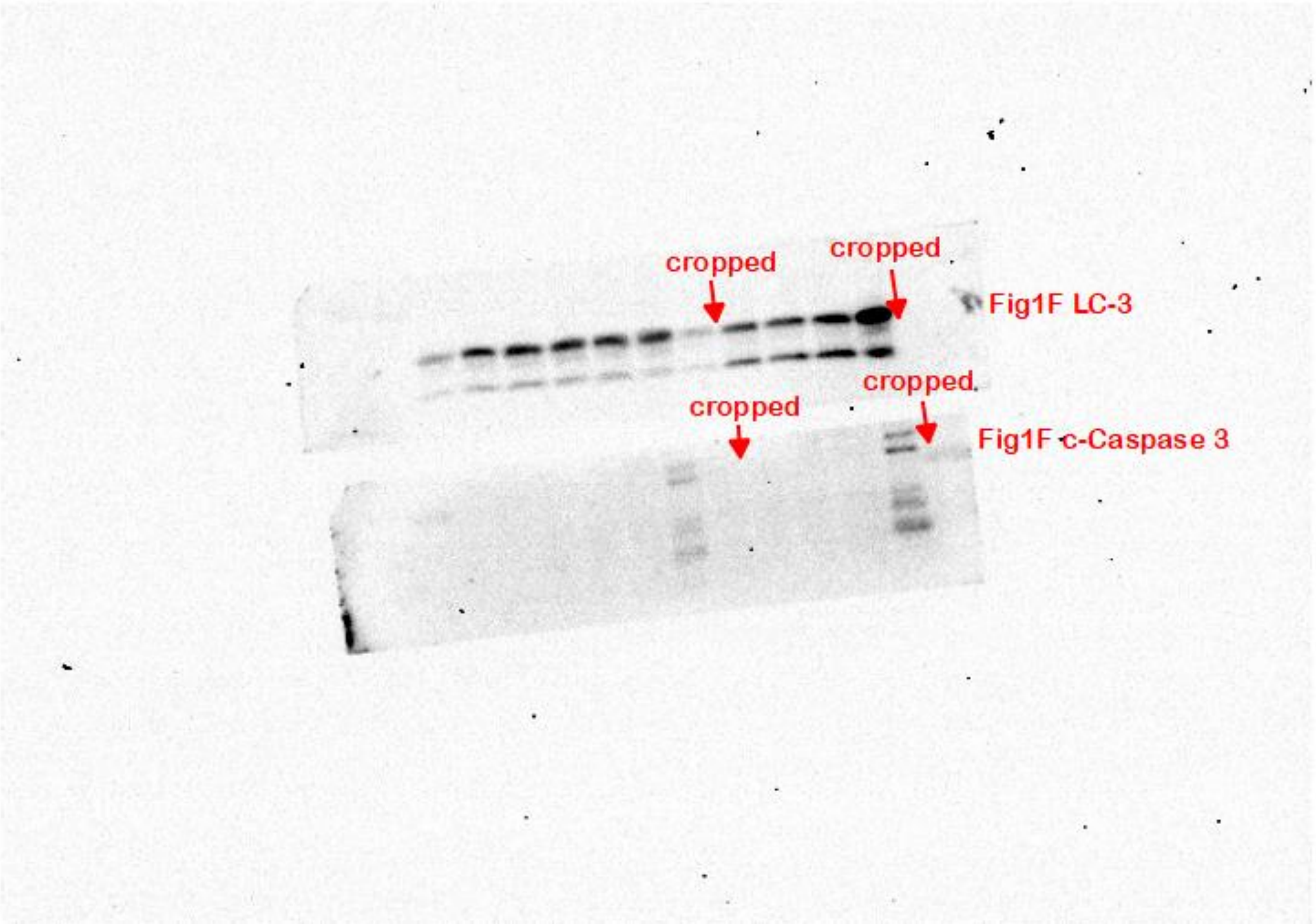

Supplementary Figure 2

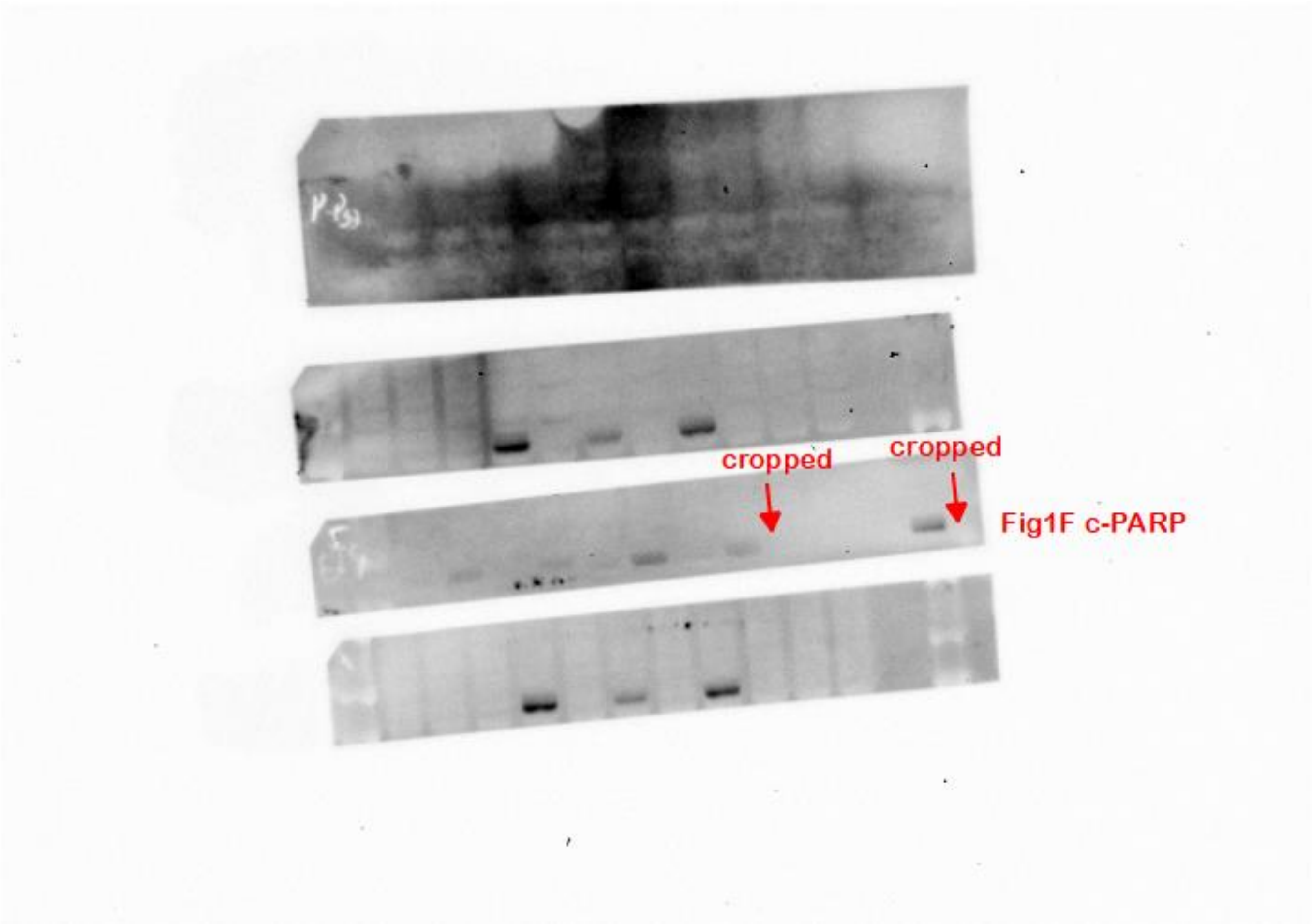

Supplementary Figure 3

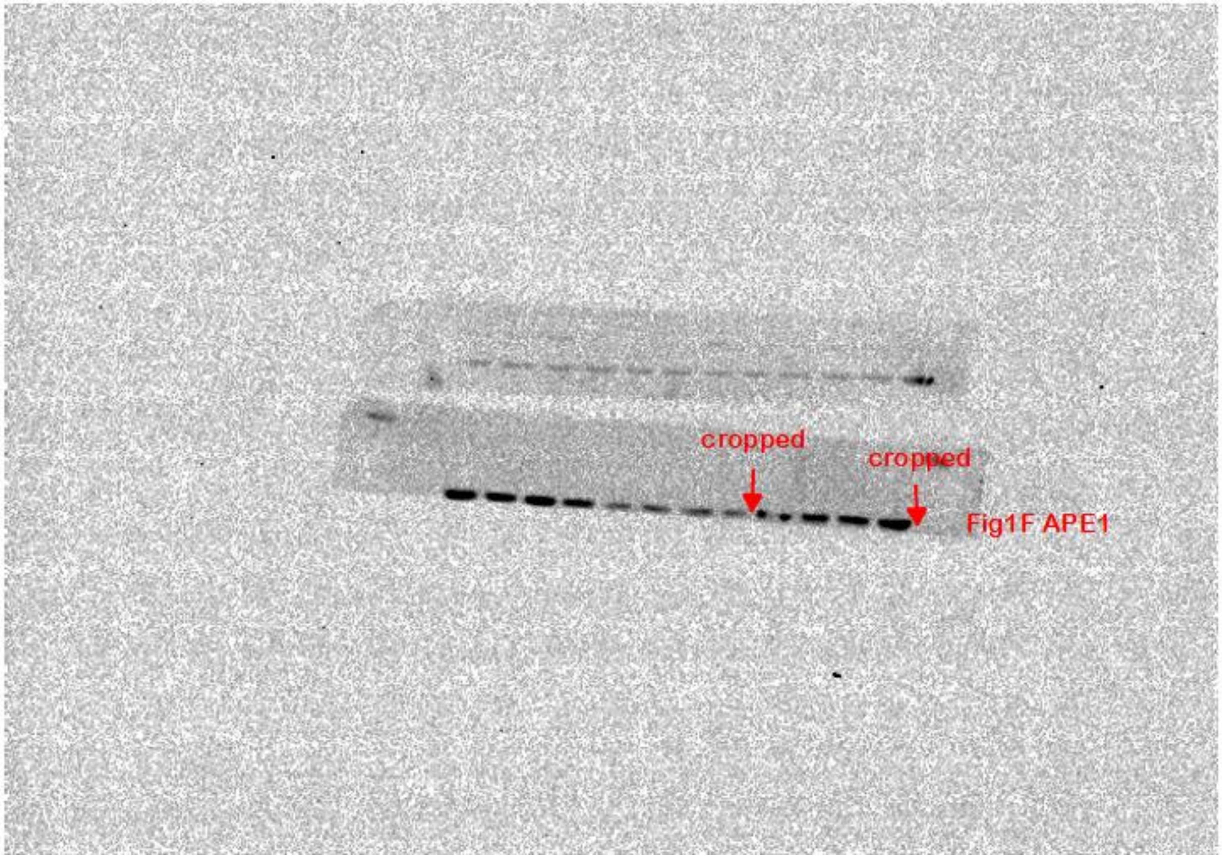

Supplementary Figure 4

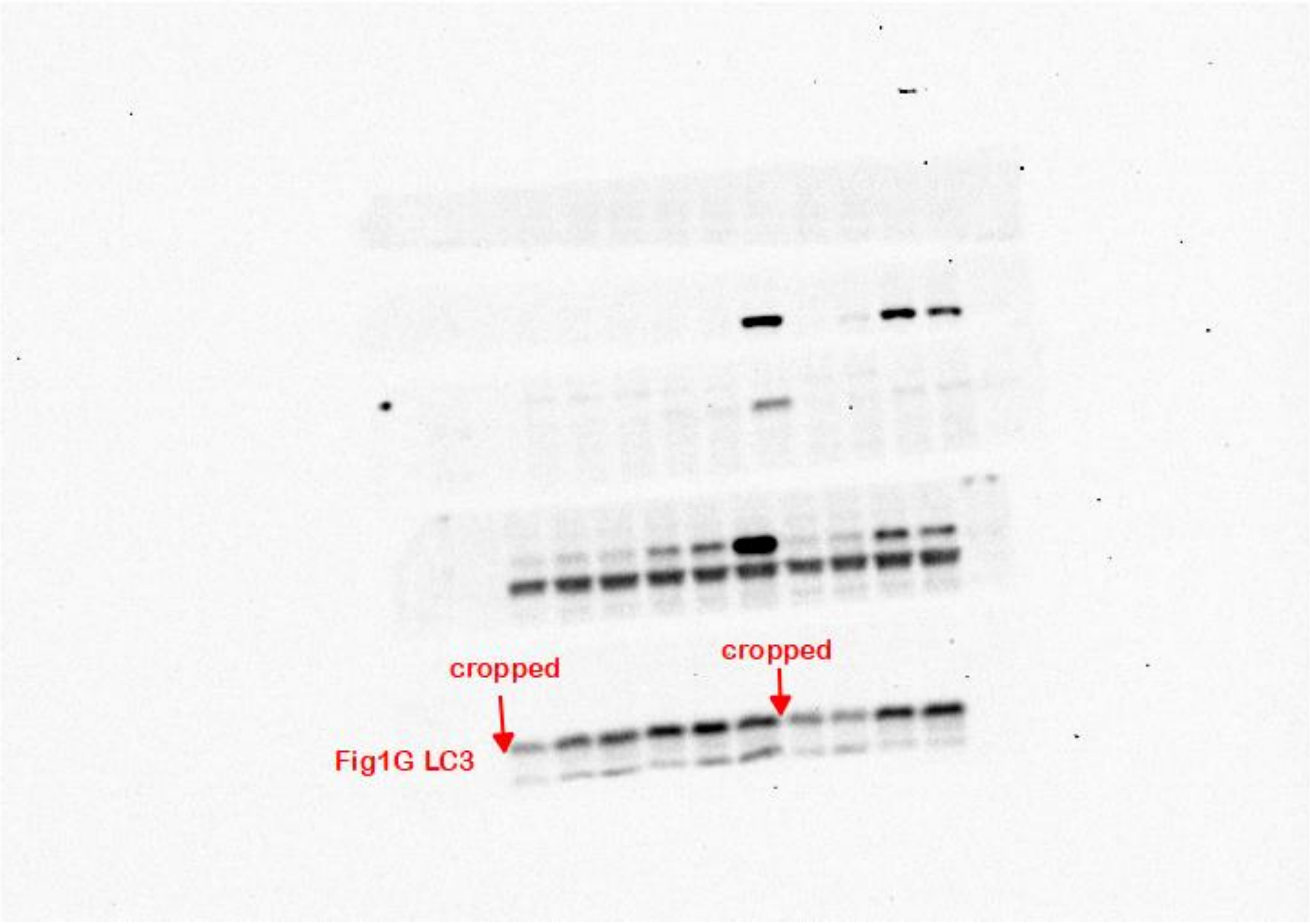

Supplementary Figure 5

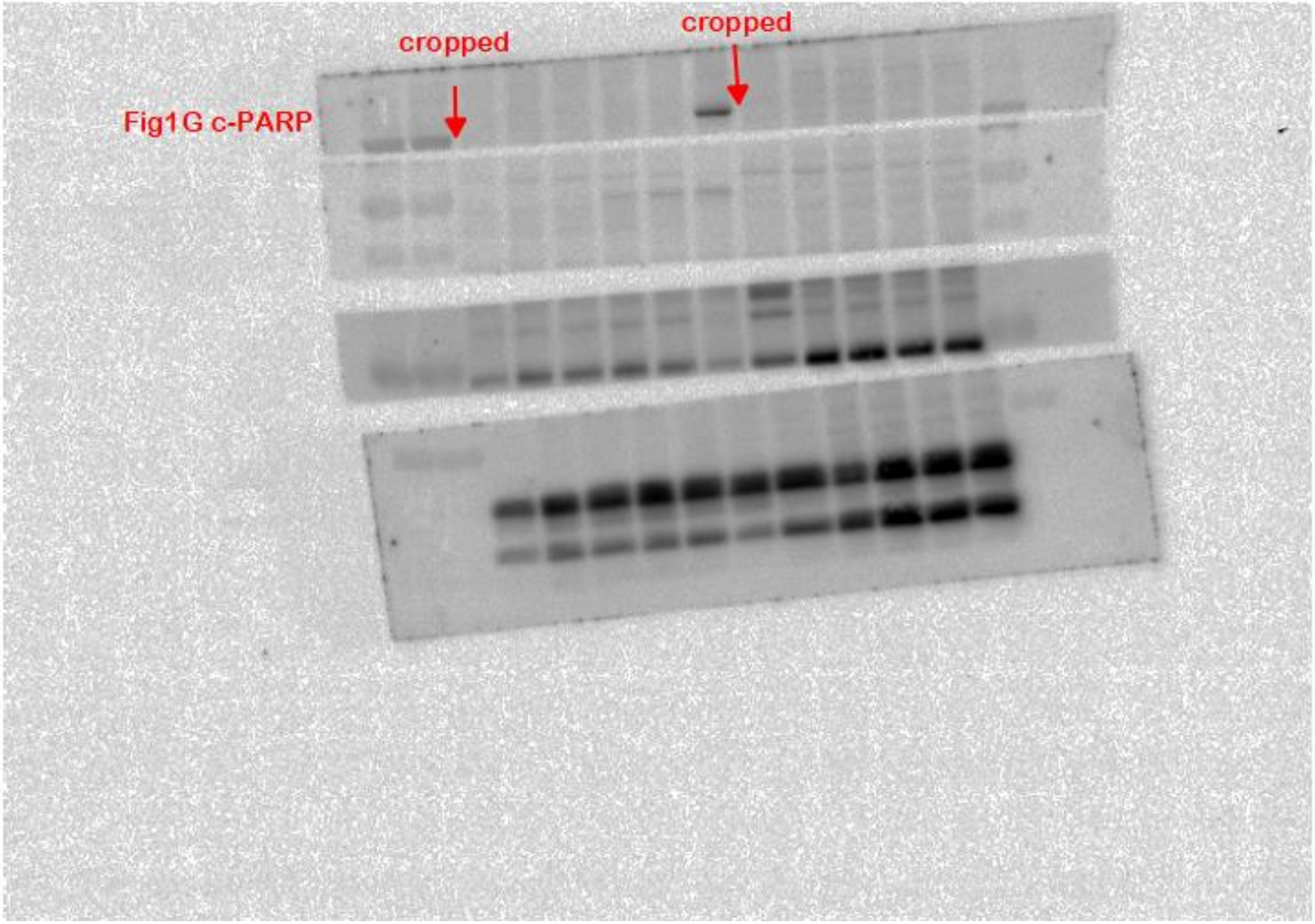

Supplementary Figure 6

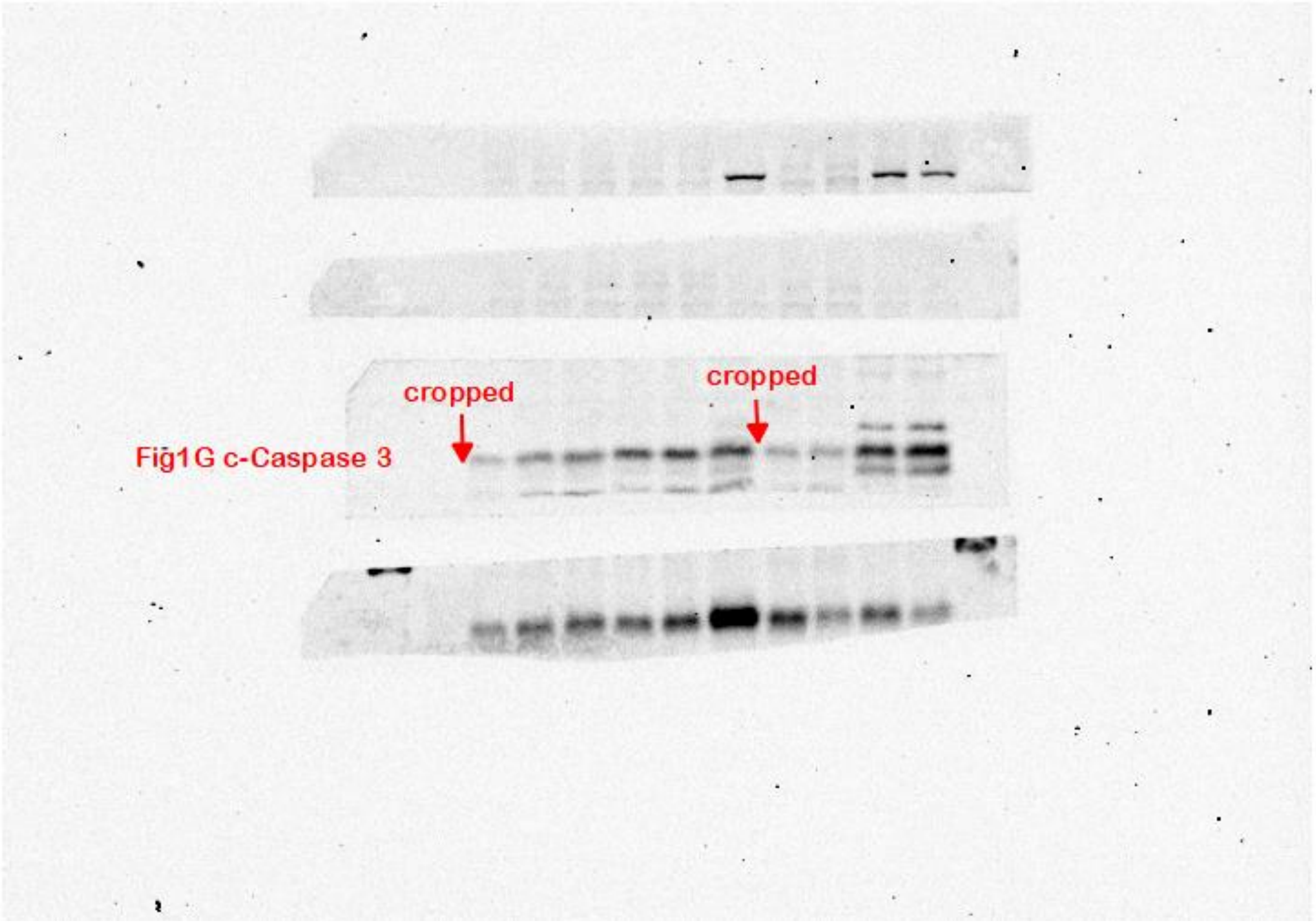

Supplementary Figure 7

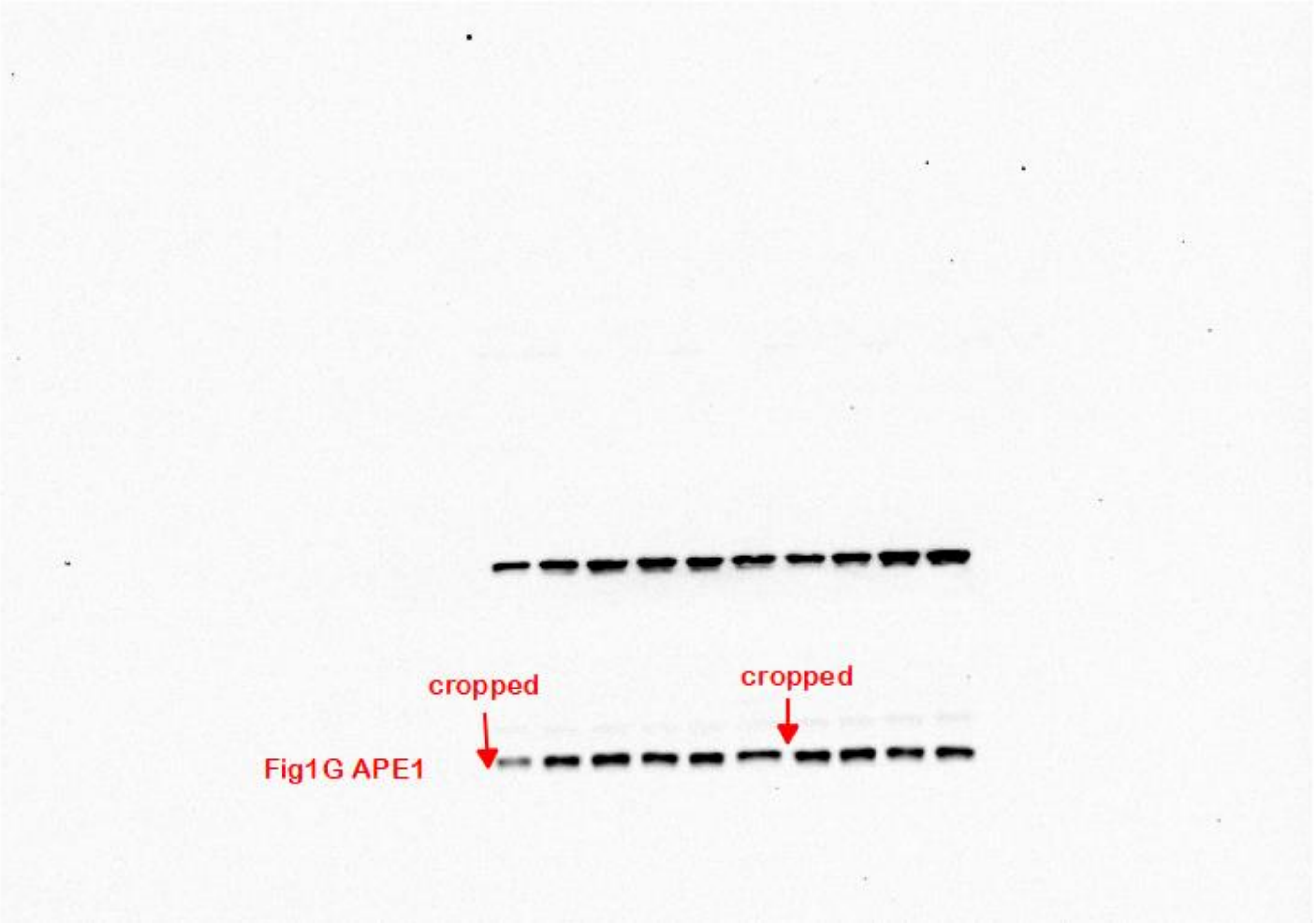

Supplementary Figure 8

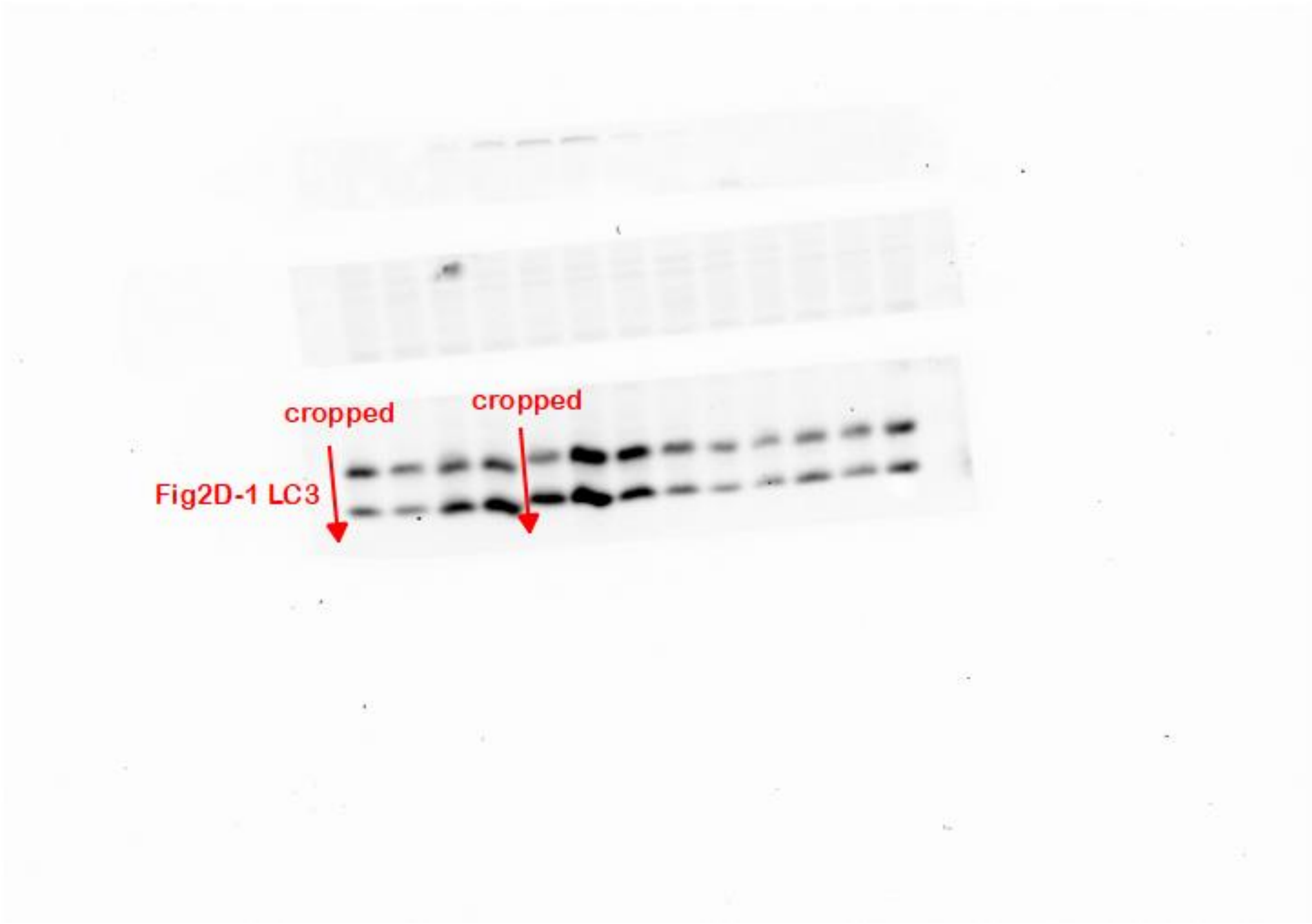

Supplementary Figure 9

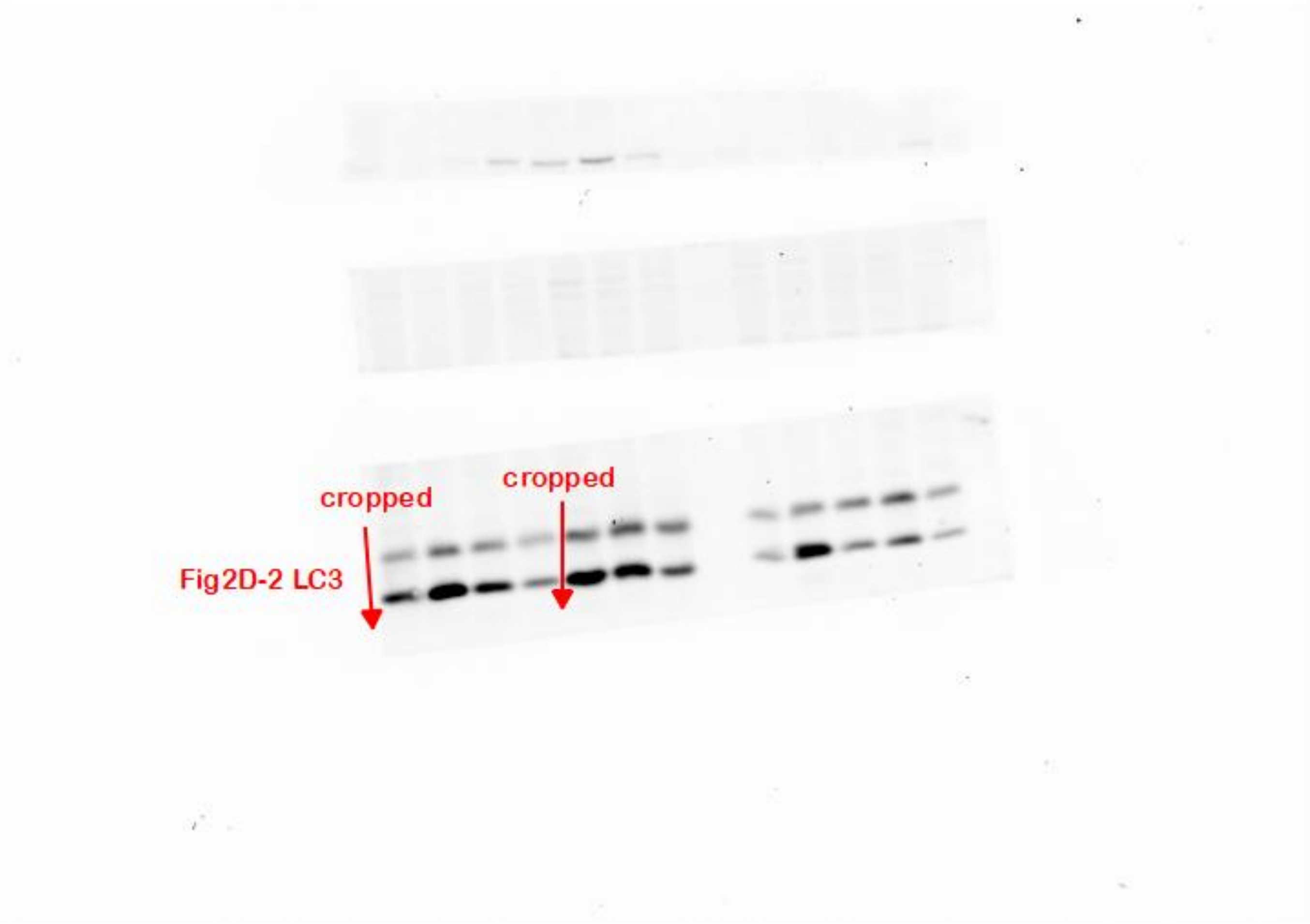

Supplementary Figure 10

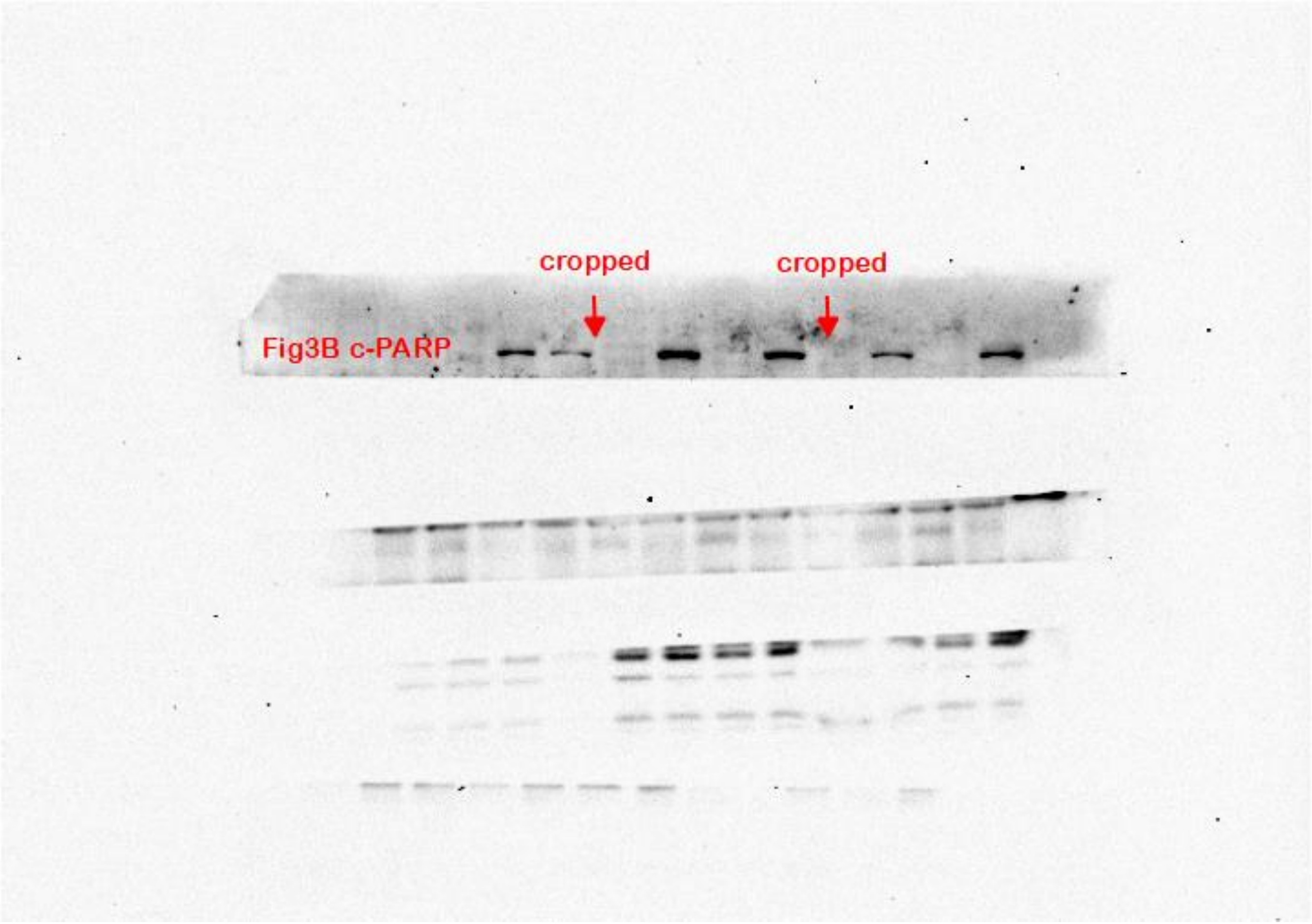

Supplementary Figure 11

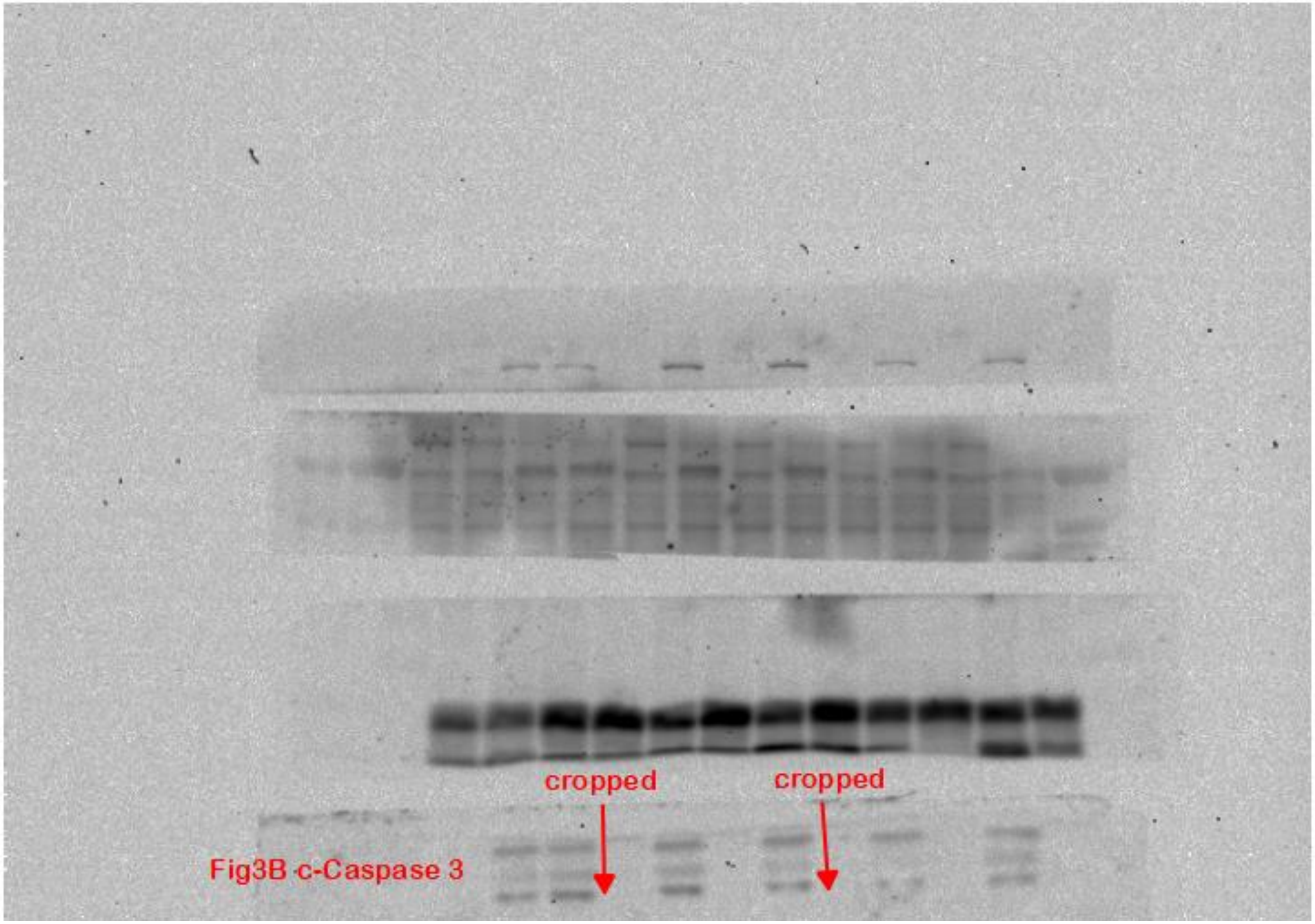

Supplementary Figure 12

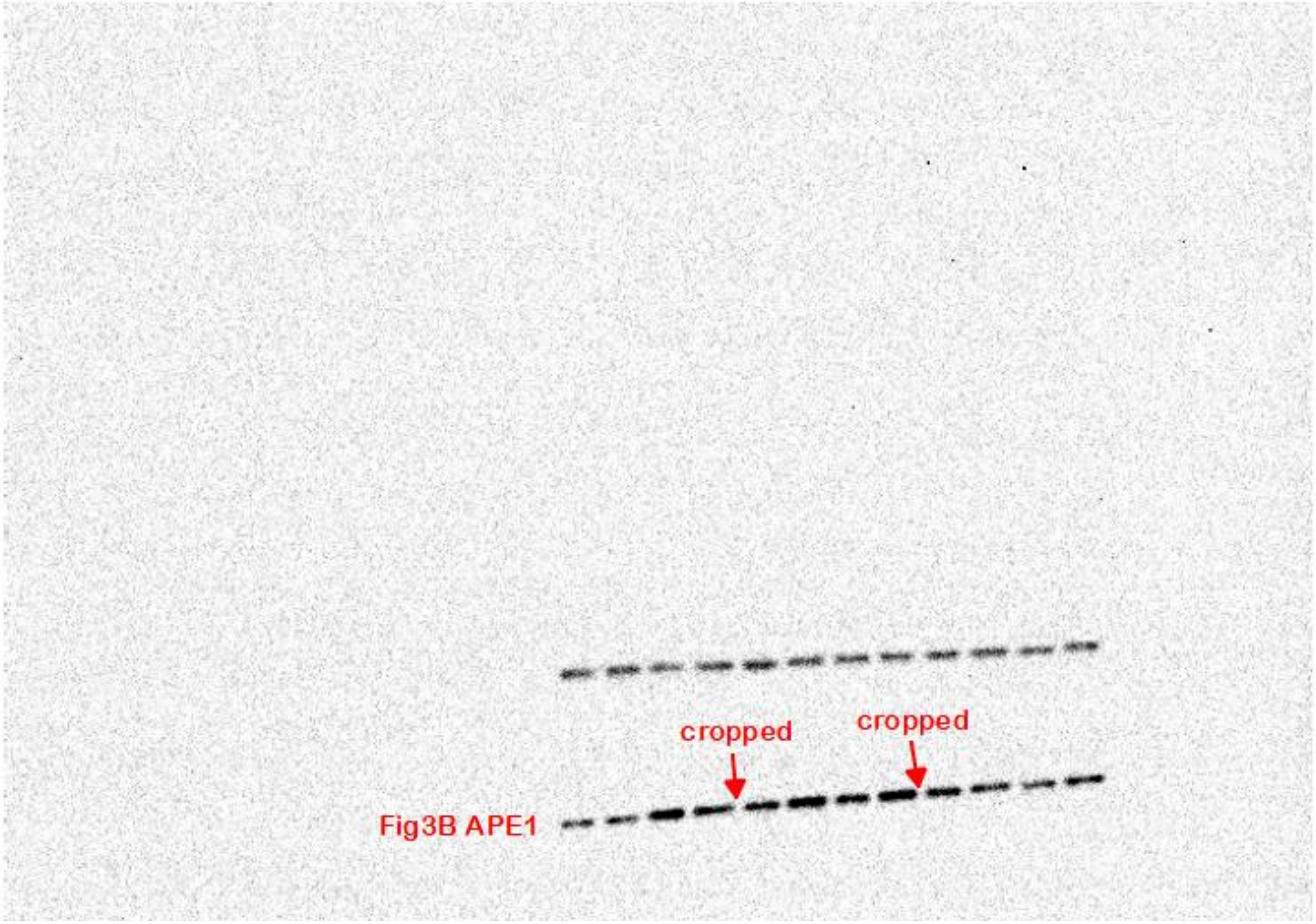

Supplementary Figure 13

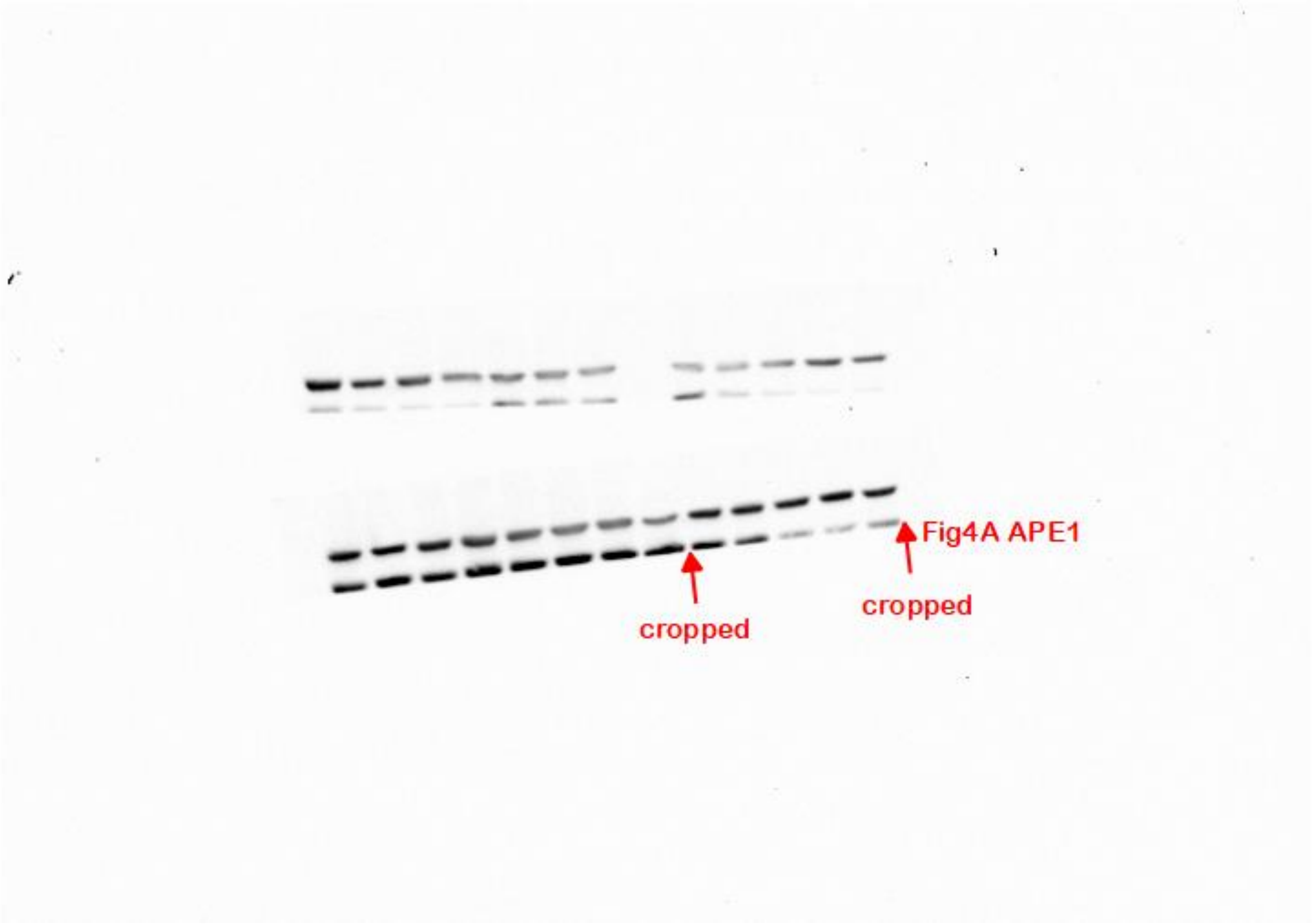

Supplementary Figure 14

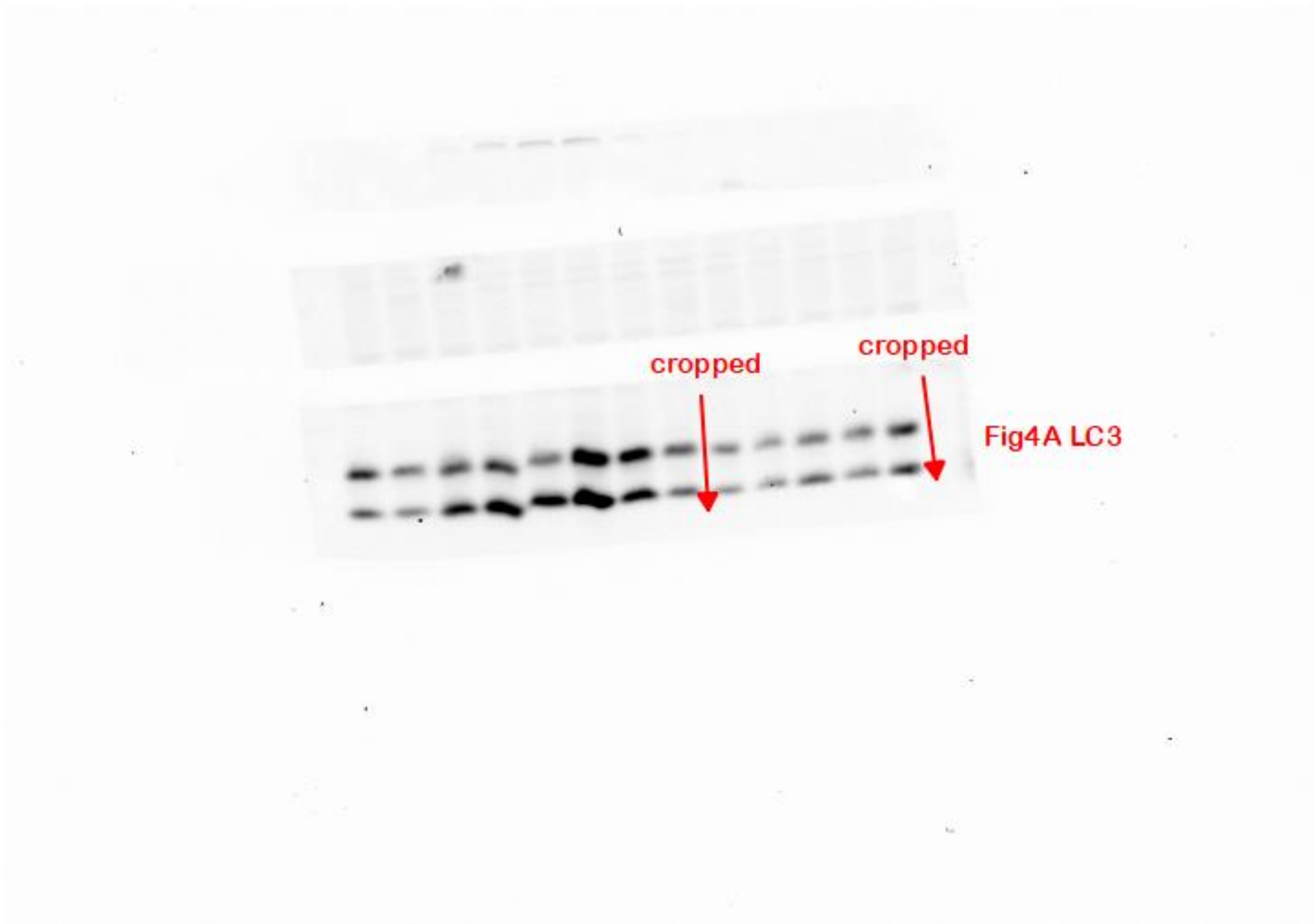

Supplementary Figure 15

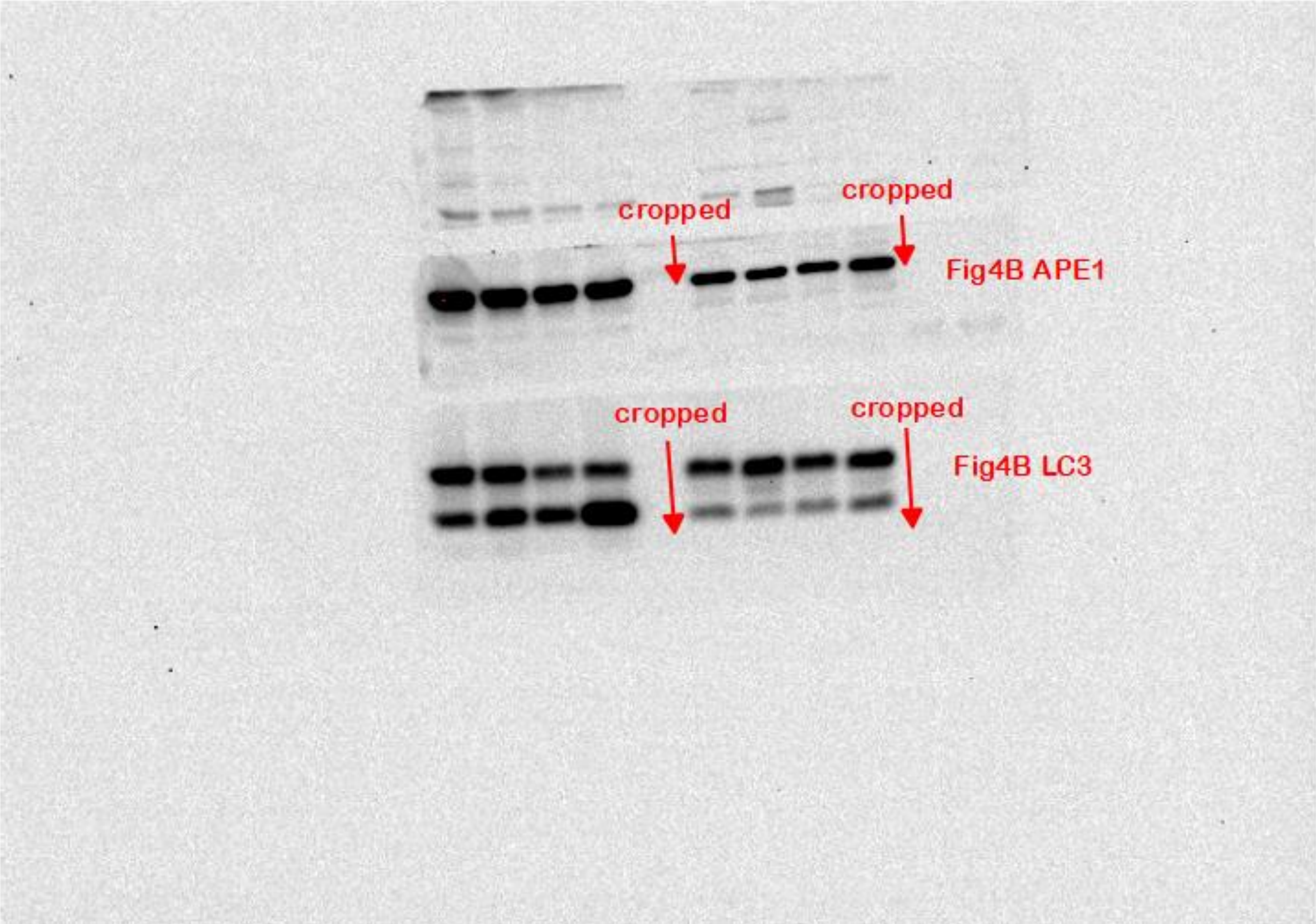

Supplementary Figure 16

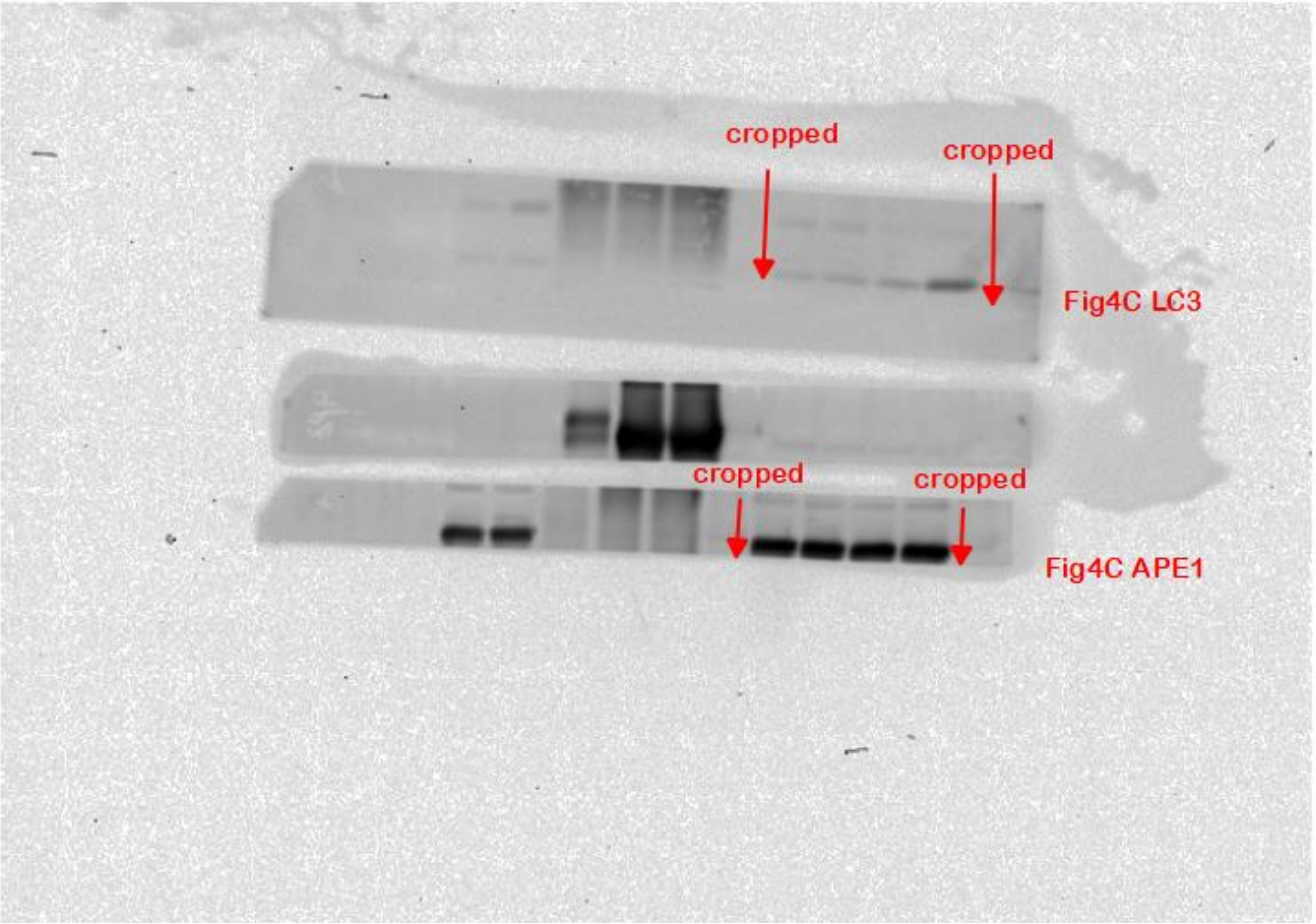

Supplementary Figure 17

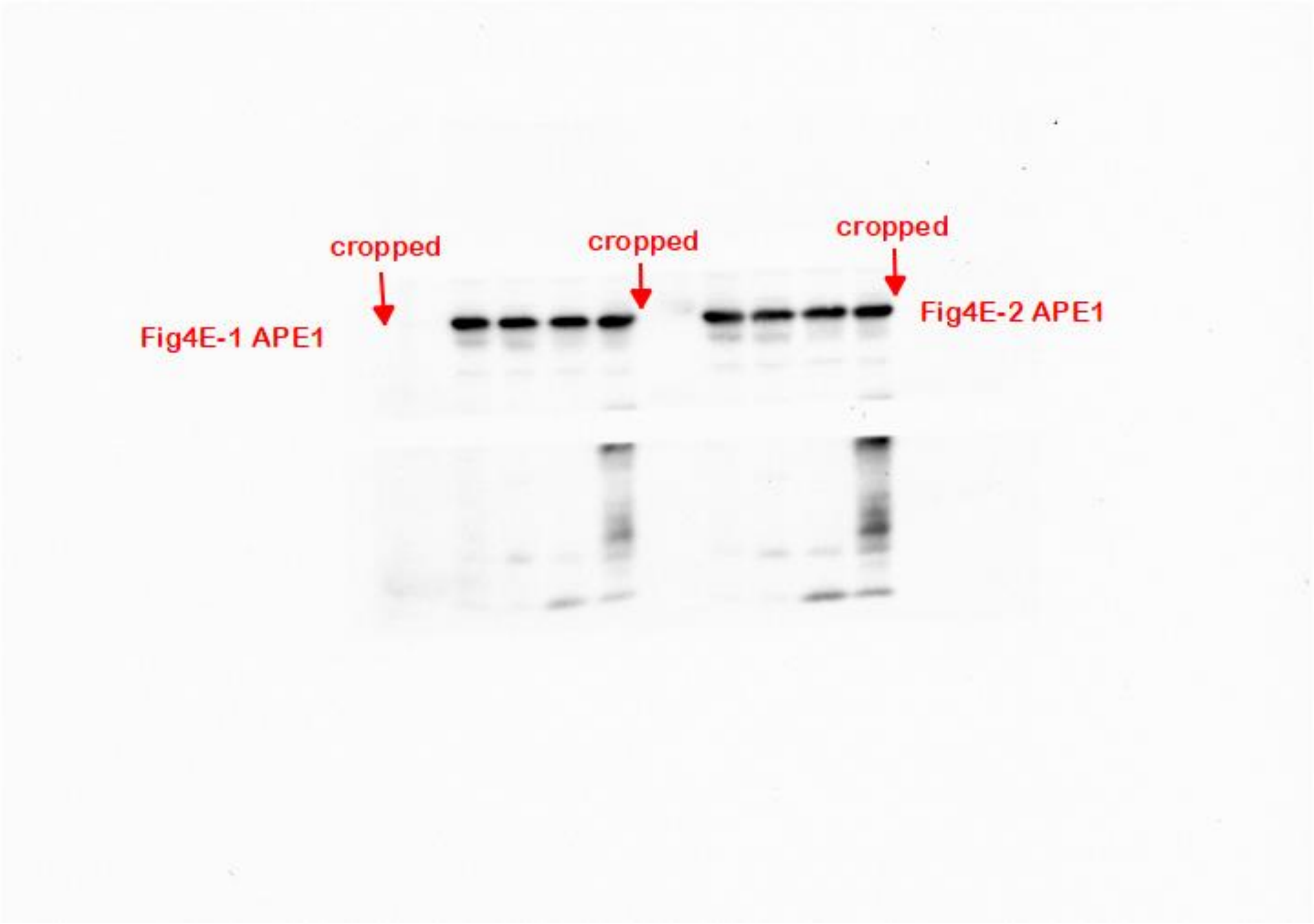

Supplementary Figure 18

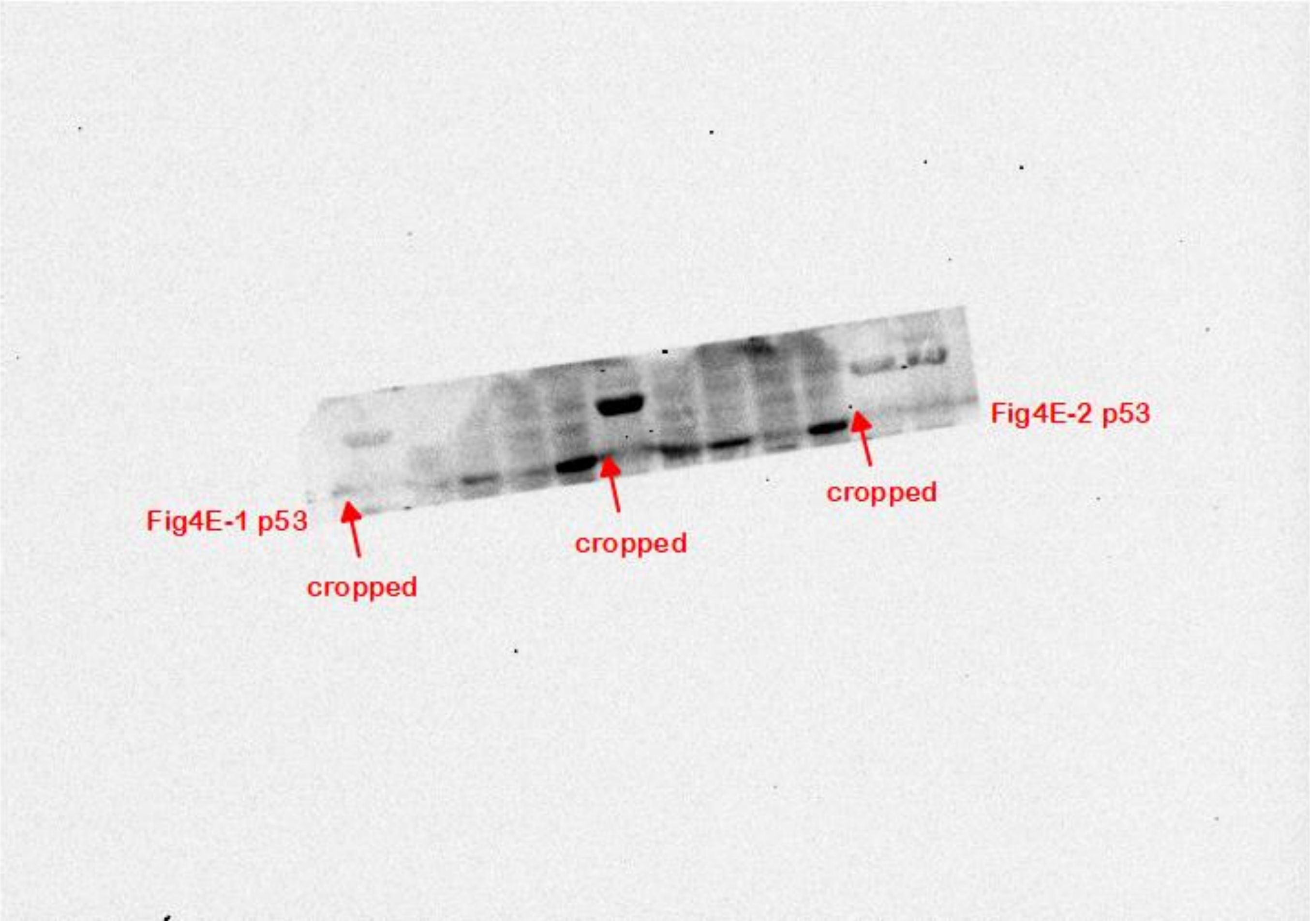

Supplementary Figure 19

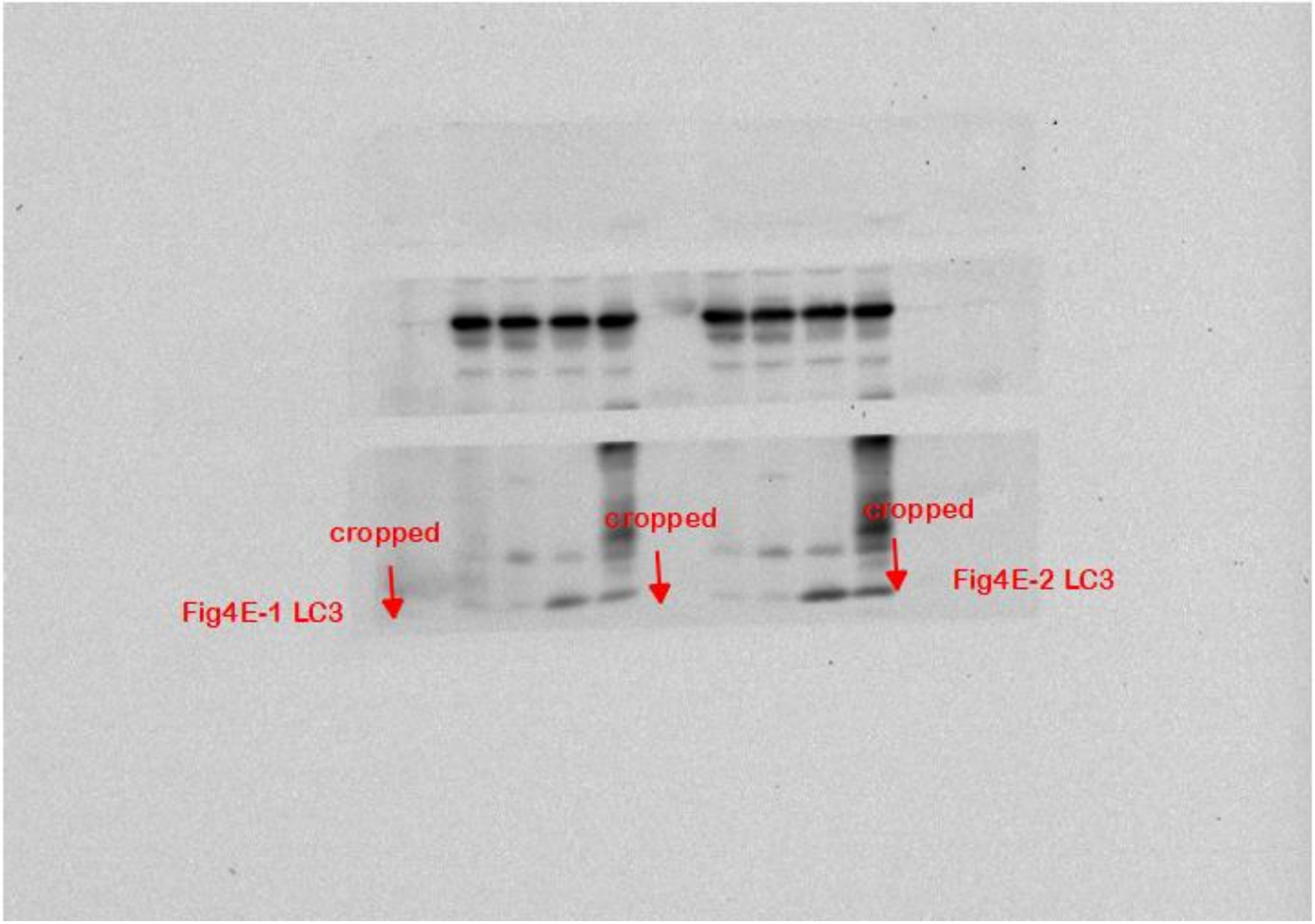

Supplementary Figure 20

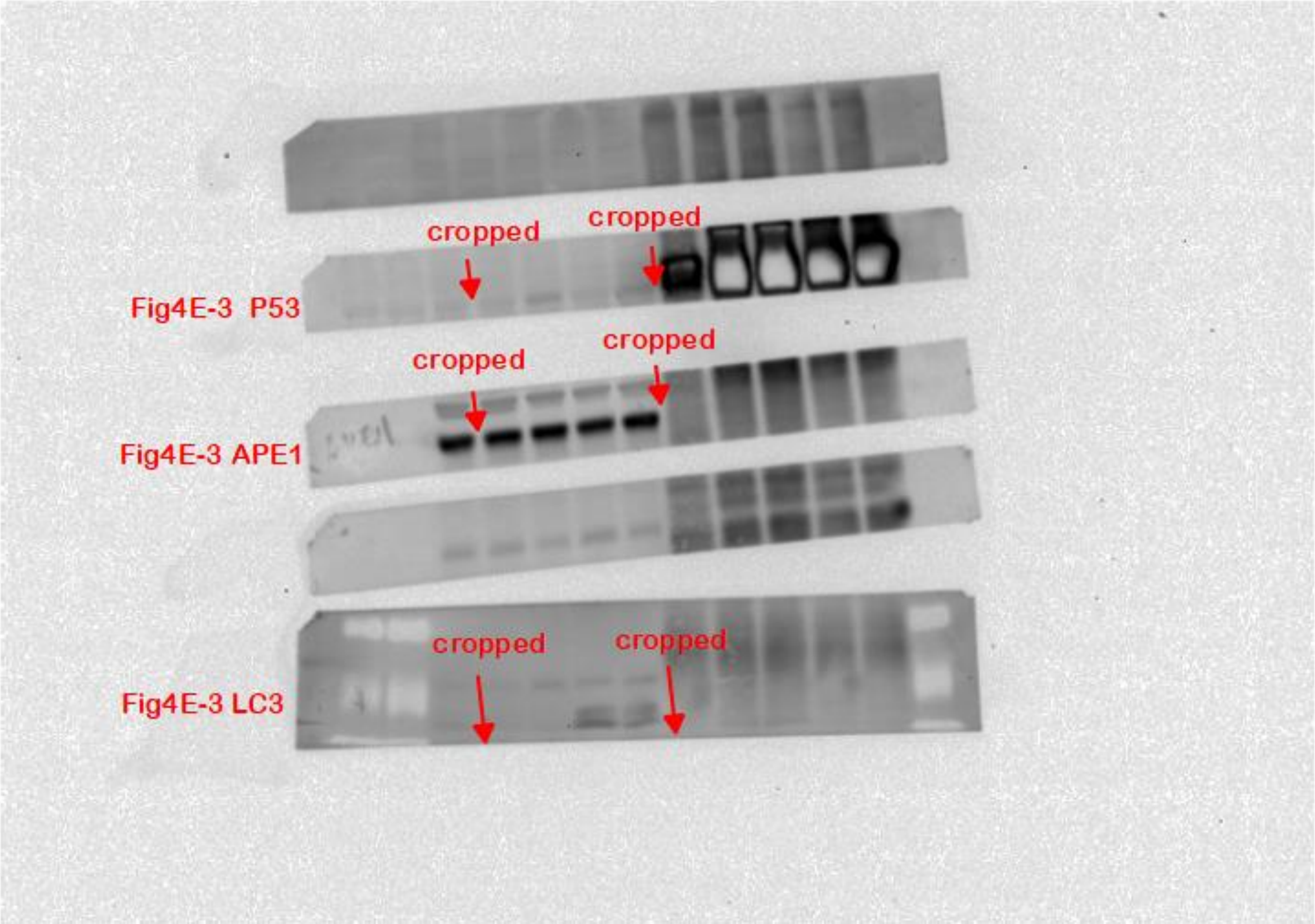

Supplementary Figure 21

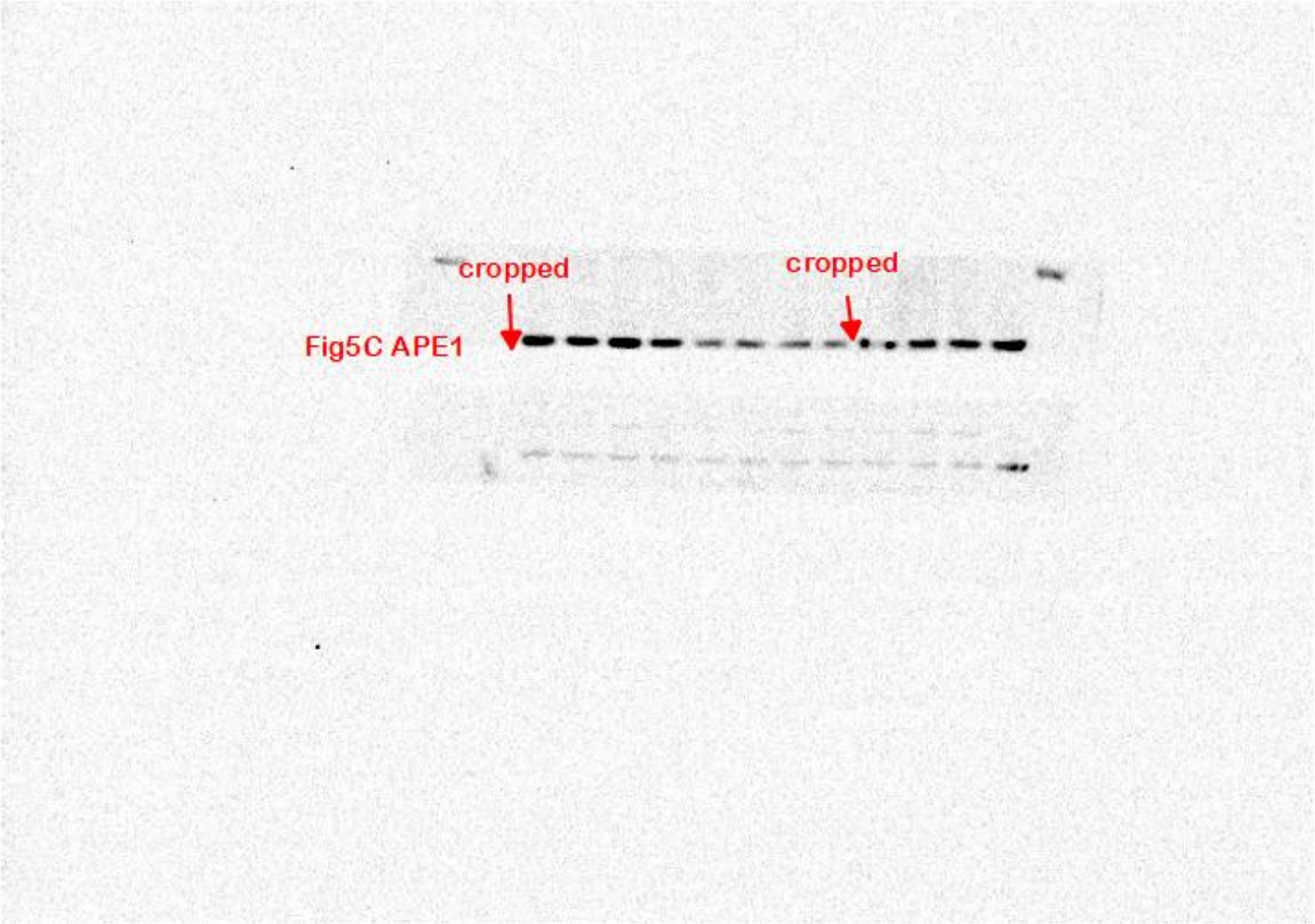

Supplementary Figure 22

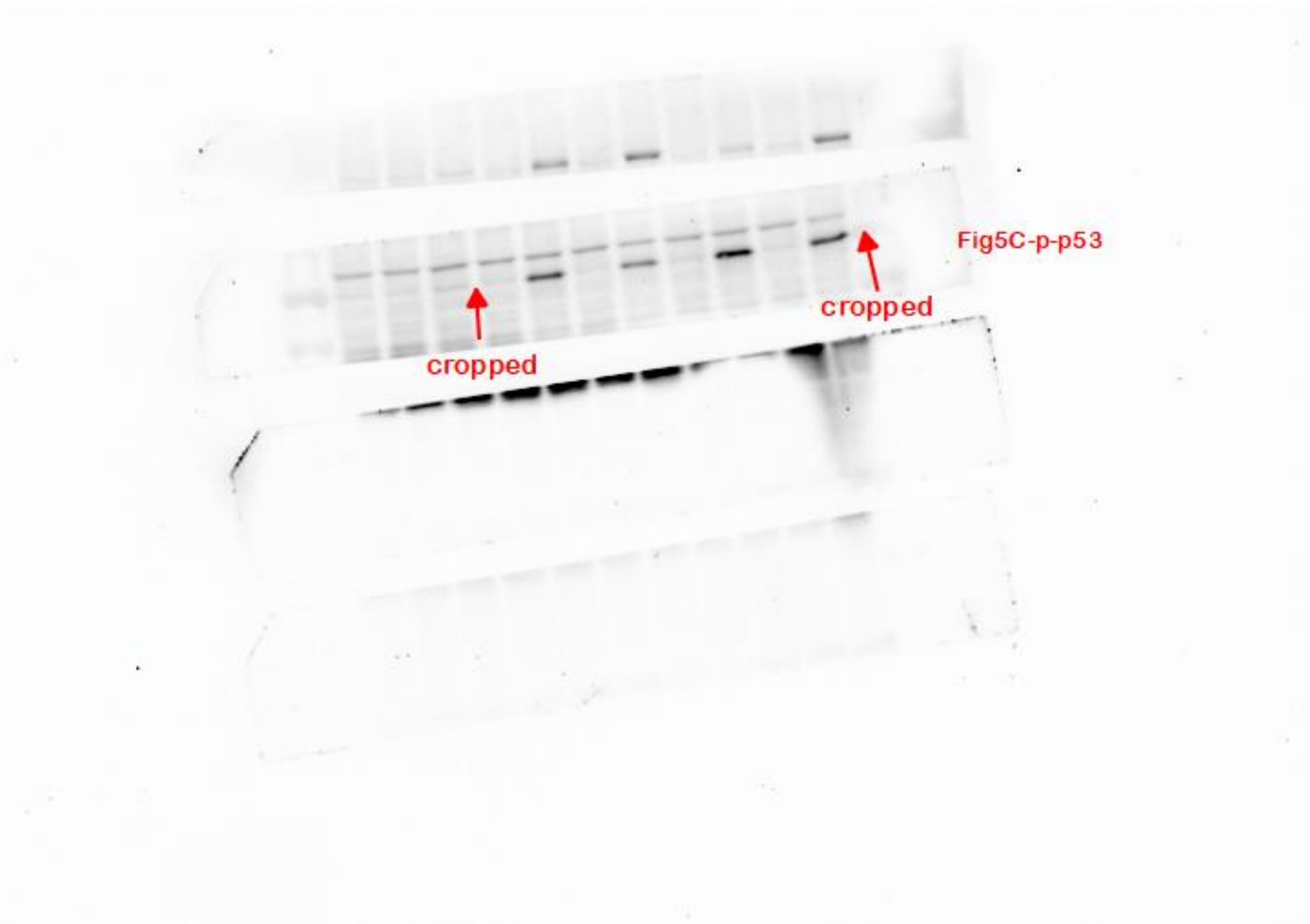

Supplementary Figure 23

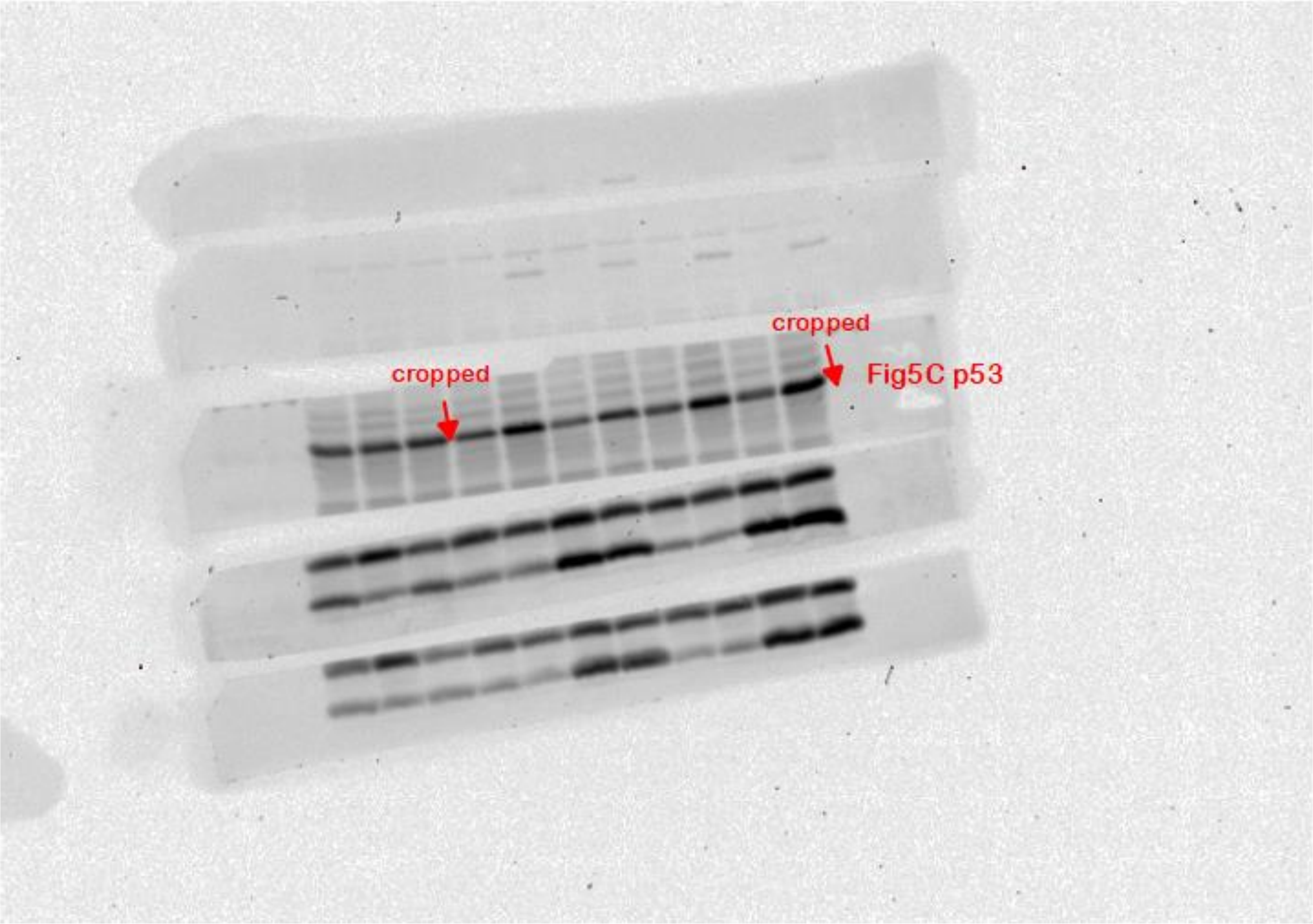

Supplementary Figure 24

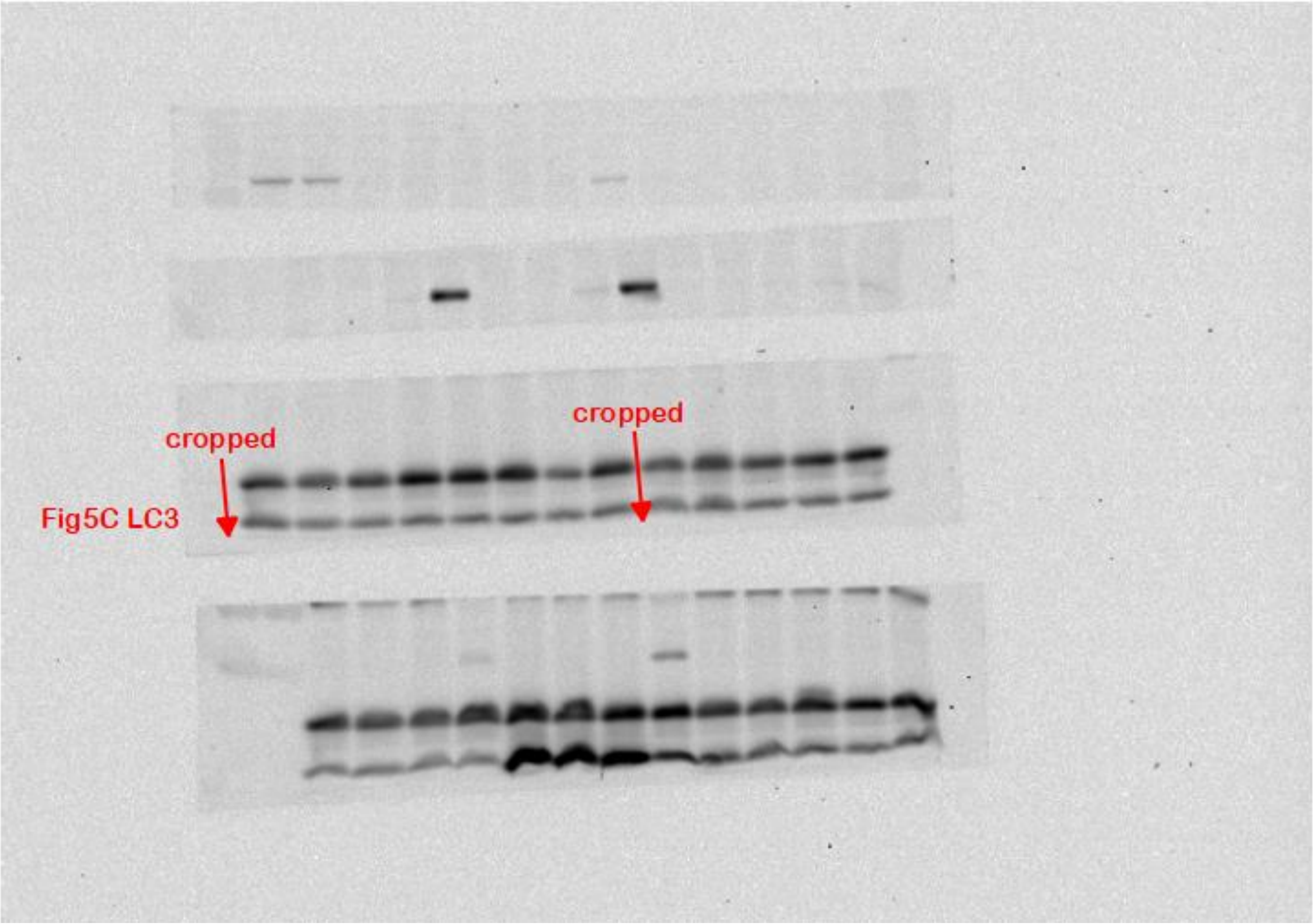

Supplementary Figure 25

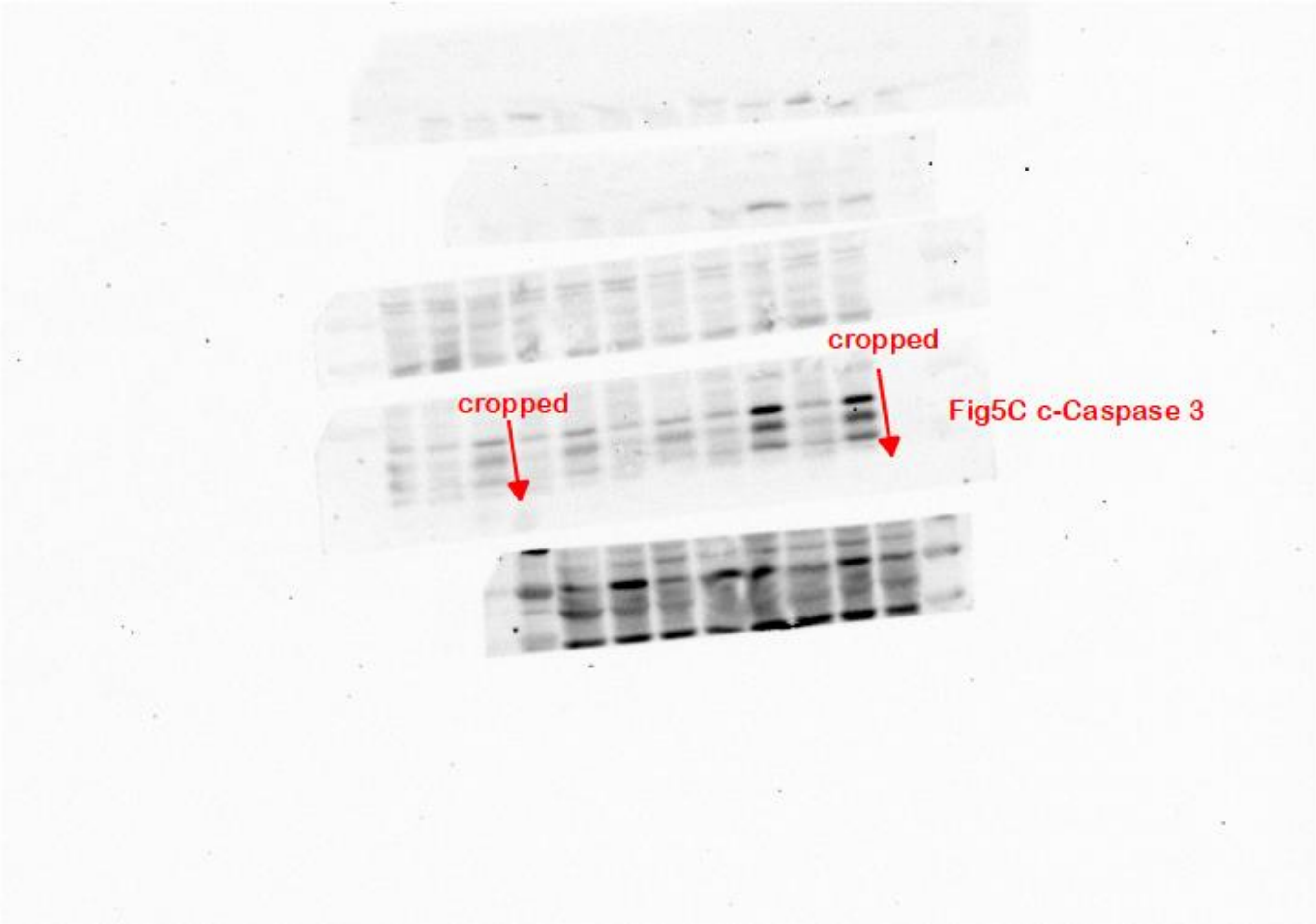

Supplementary Figure 26

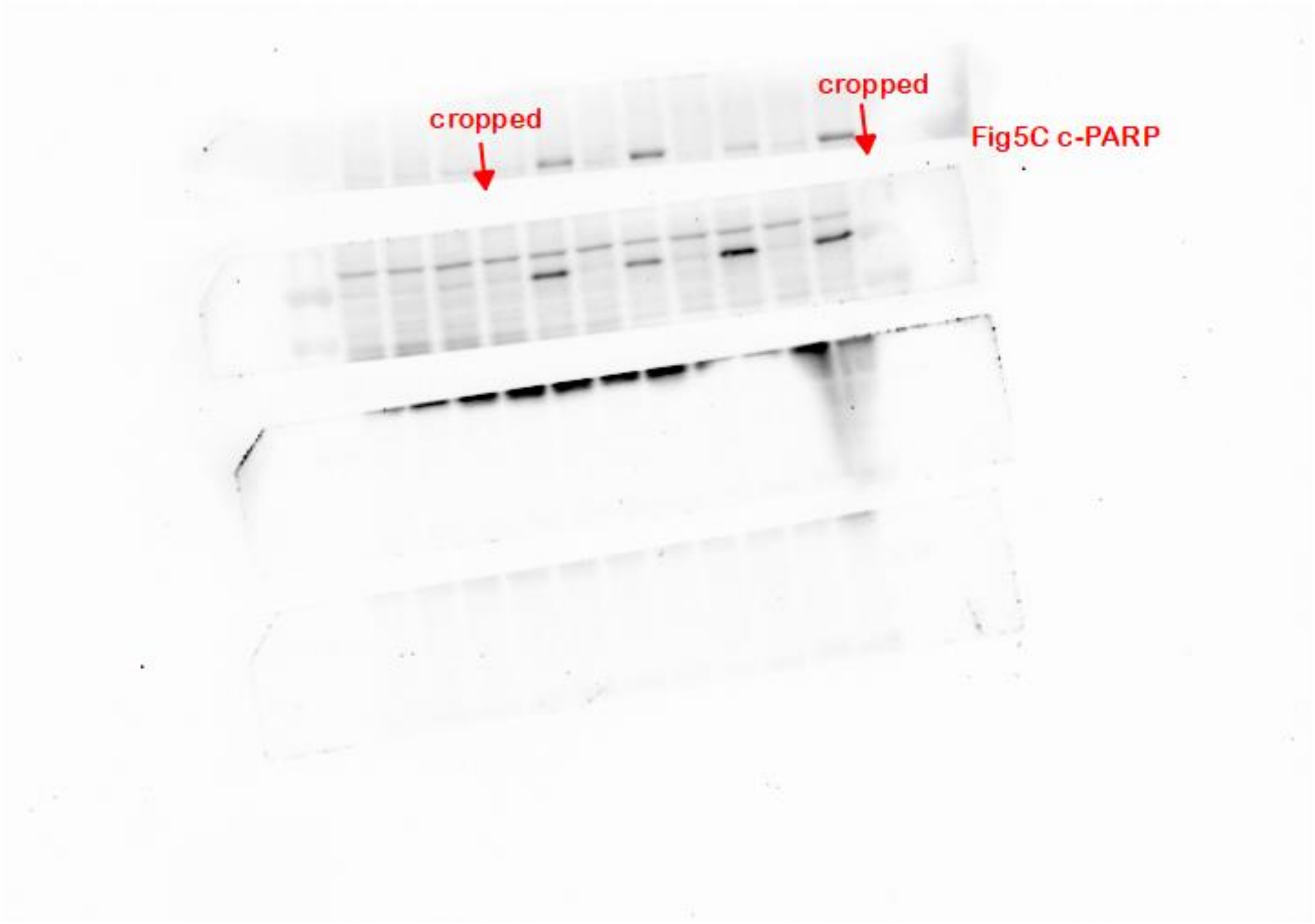

Supplementary Figure 27

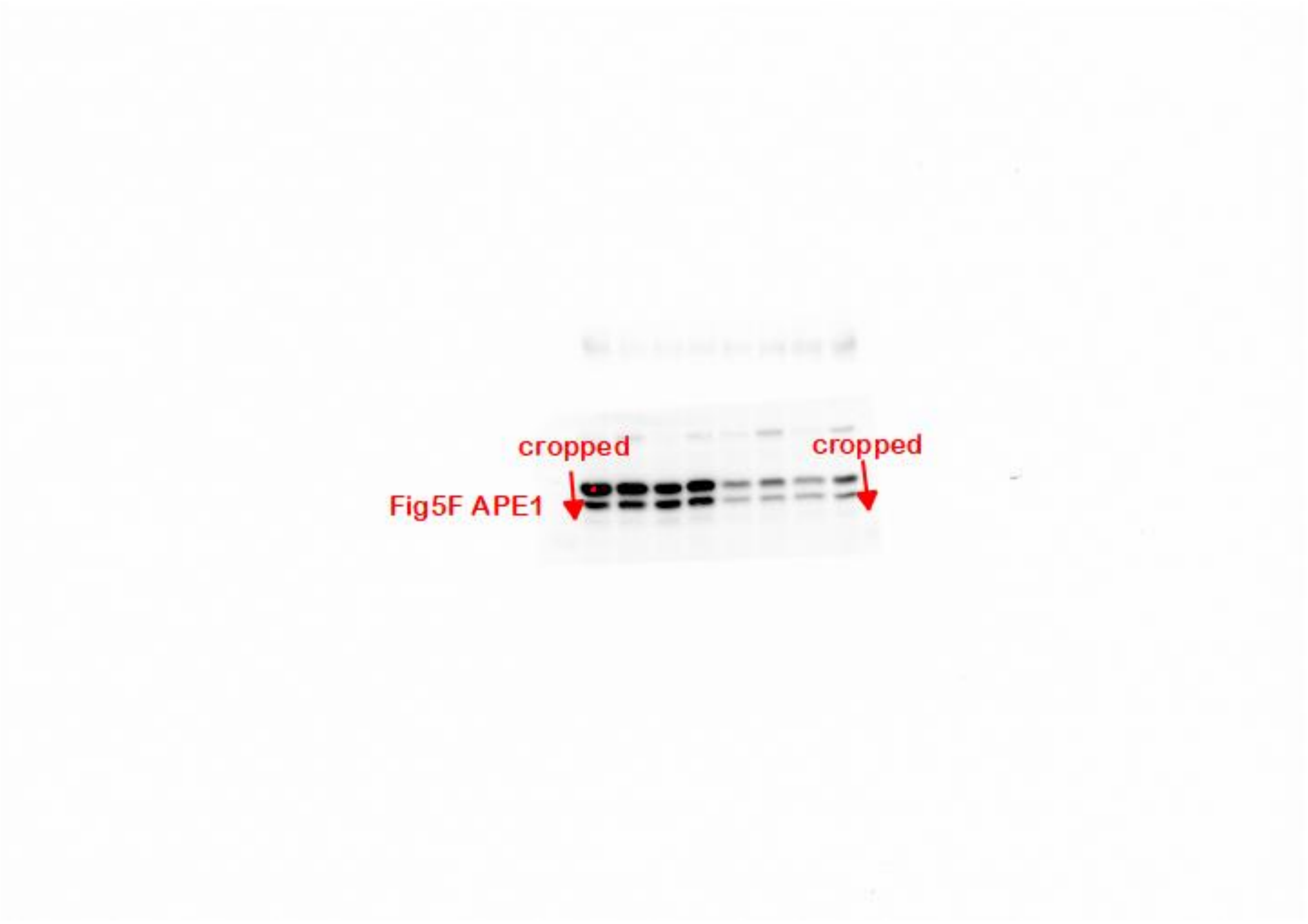

Supplementary Figure 28

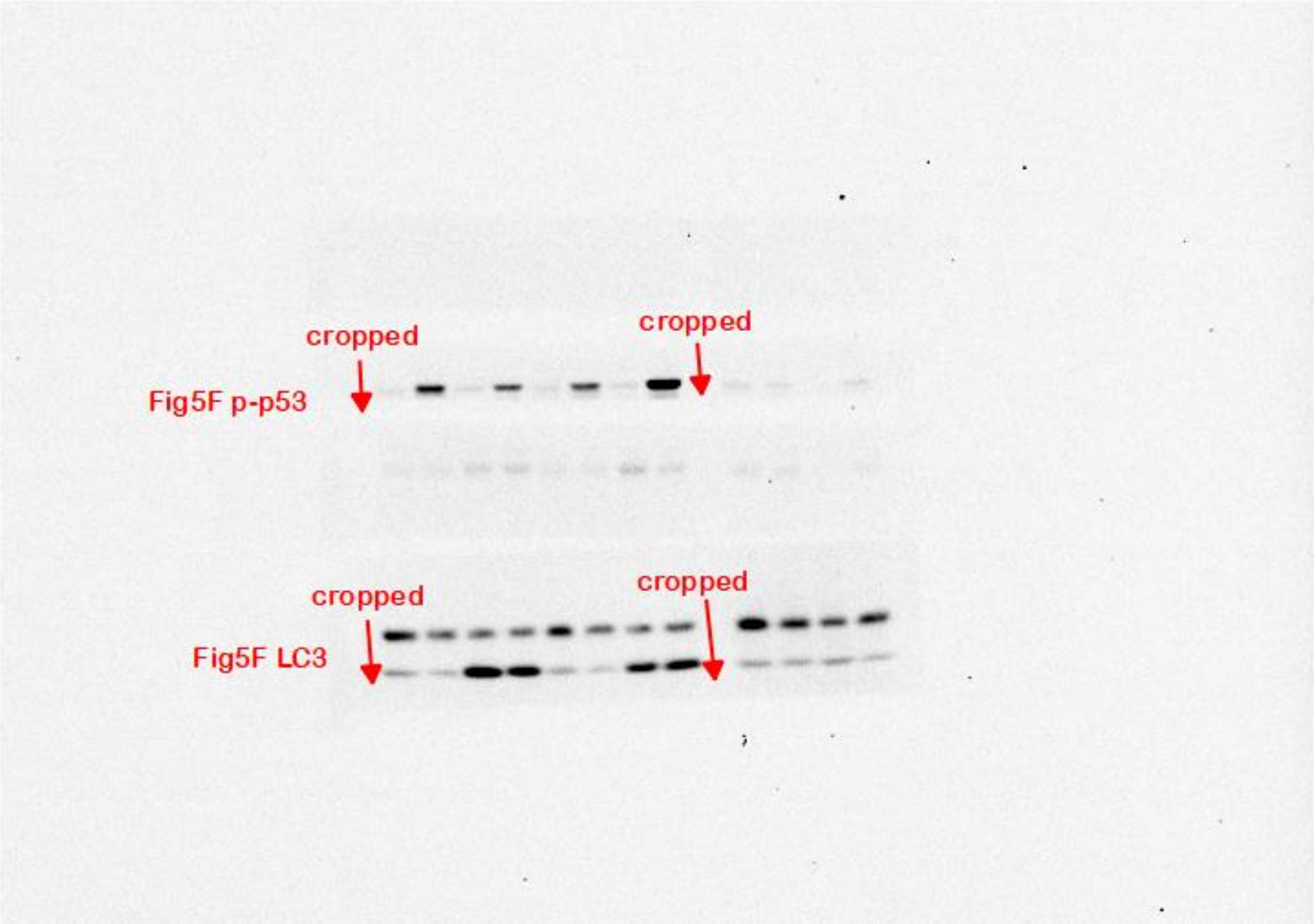

Supplementary Figure 29

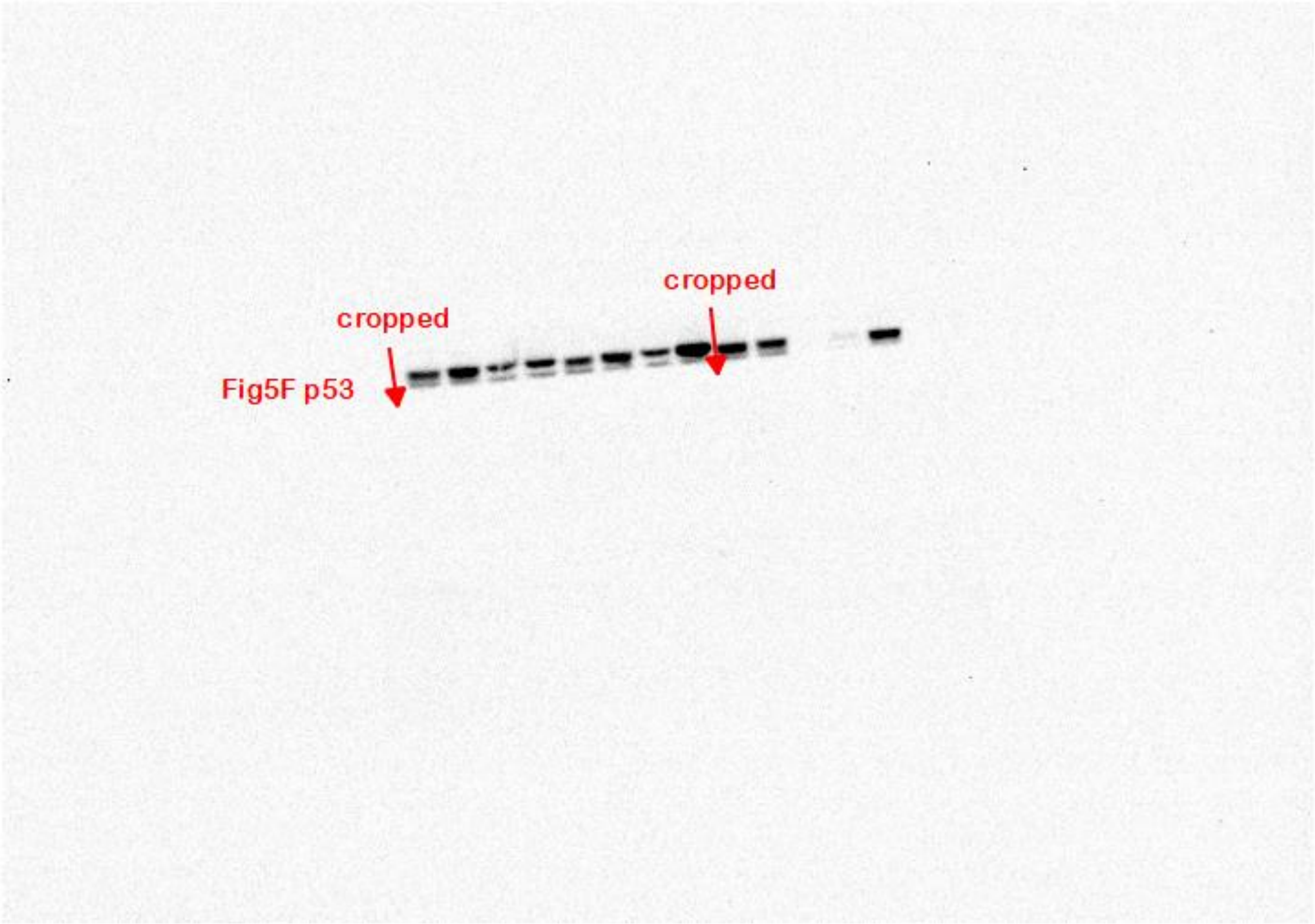

Supplementary Figure 30

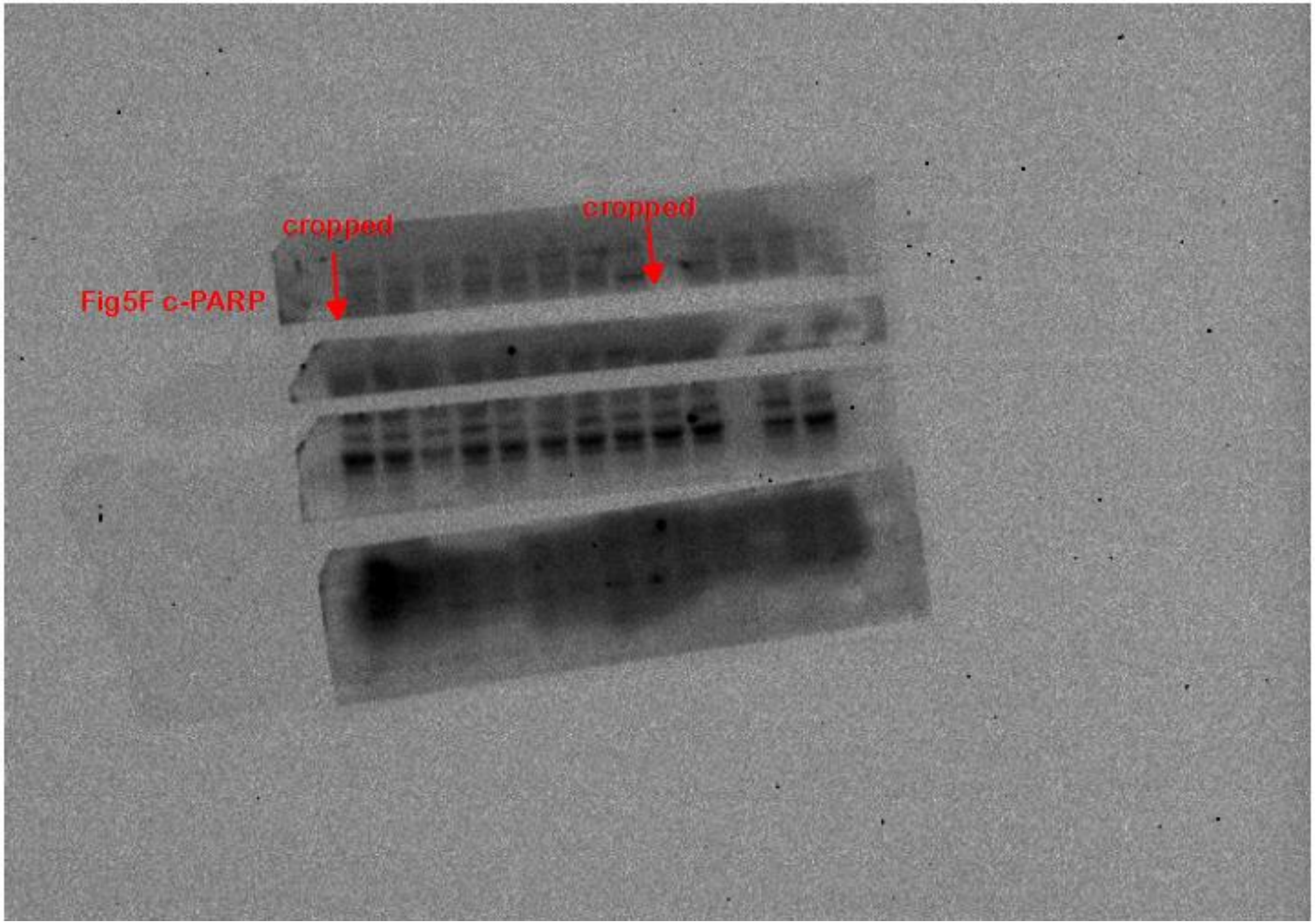

Supplementary Figure 31

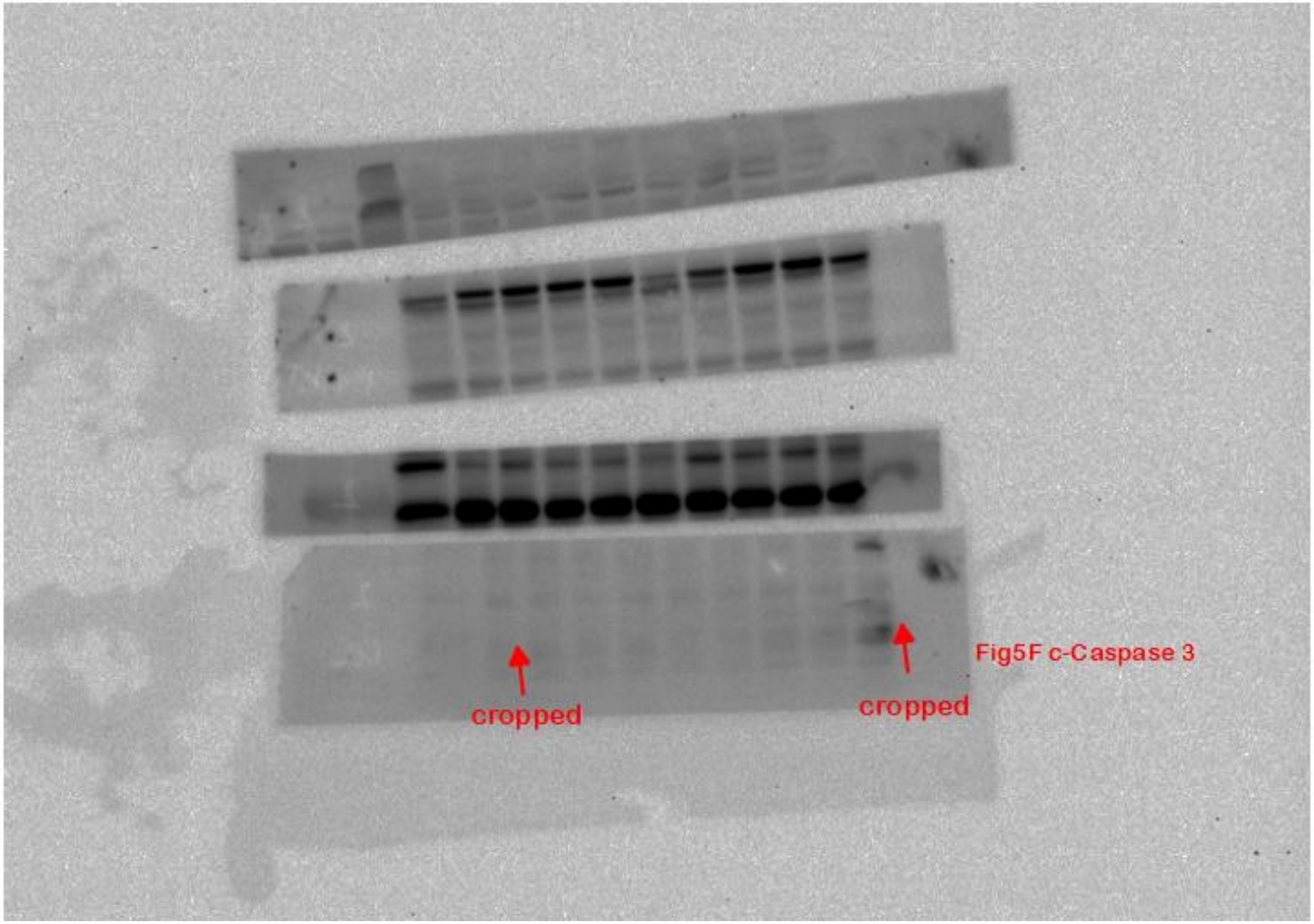

Supplement: Supplementary file 1 — Additional file 1: Figure S1-S31. Original gels and blot images. Image Lab 3.0 software (Bio-Rad, USA) was used to analyse the blots. The cropping of the blots was labelled with the symbol of “↓”. Corresponding uncropped full-length blots are presented in Supplementary Figure [1–31]. [file 12885_2020_7111_MOESM1_ESM.pdf]
