# Supplementary figures and images for: Proteomics reveals a therapeutic vulnerability via the combined blockade of APE1 and autophagy in lung cancer A549 cells
Source: BMC Cancer. 2020 Jul 8;20:634. doi: 10.1186/s12885-020-07111-w (PMC7346405; doi:10.1186/s12885-020-07111-w)

A

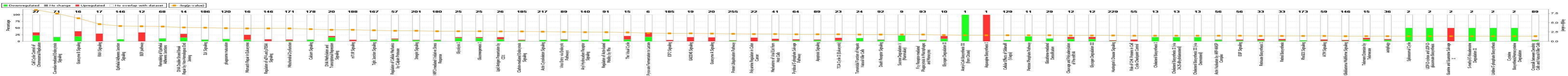

B

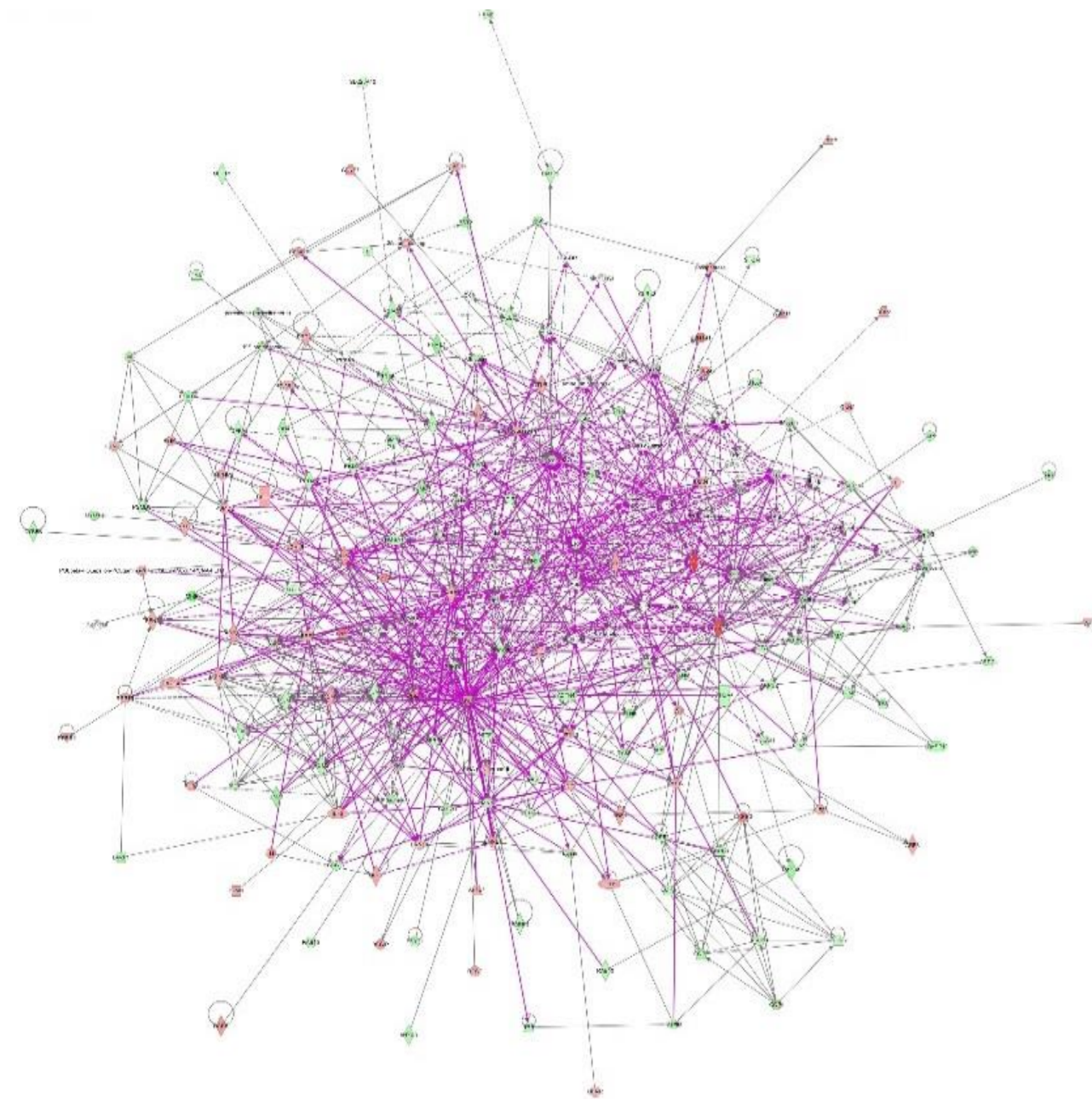

C

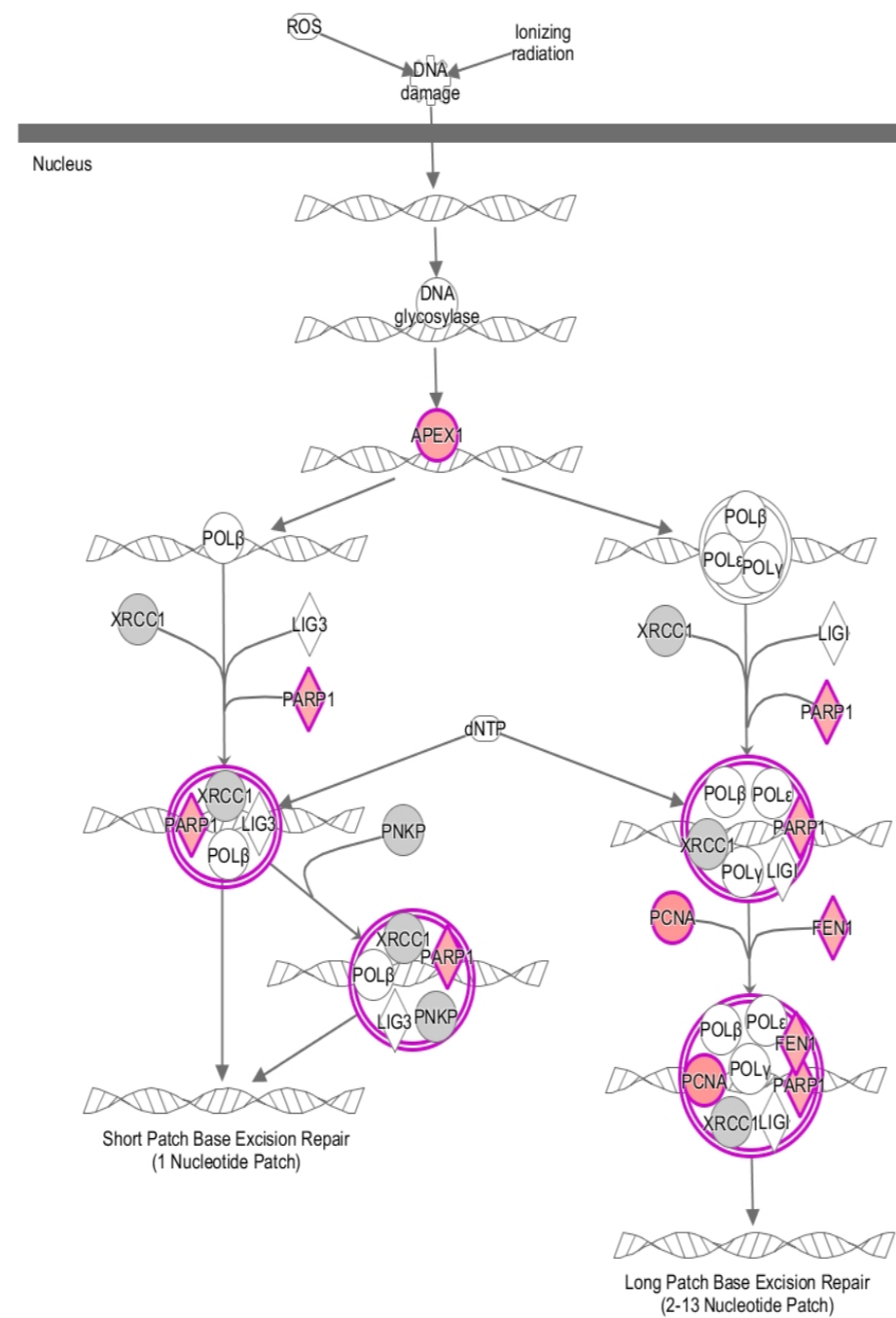

D

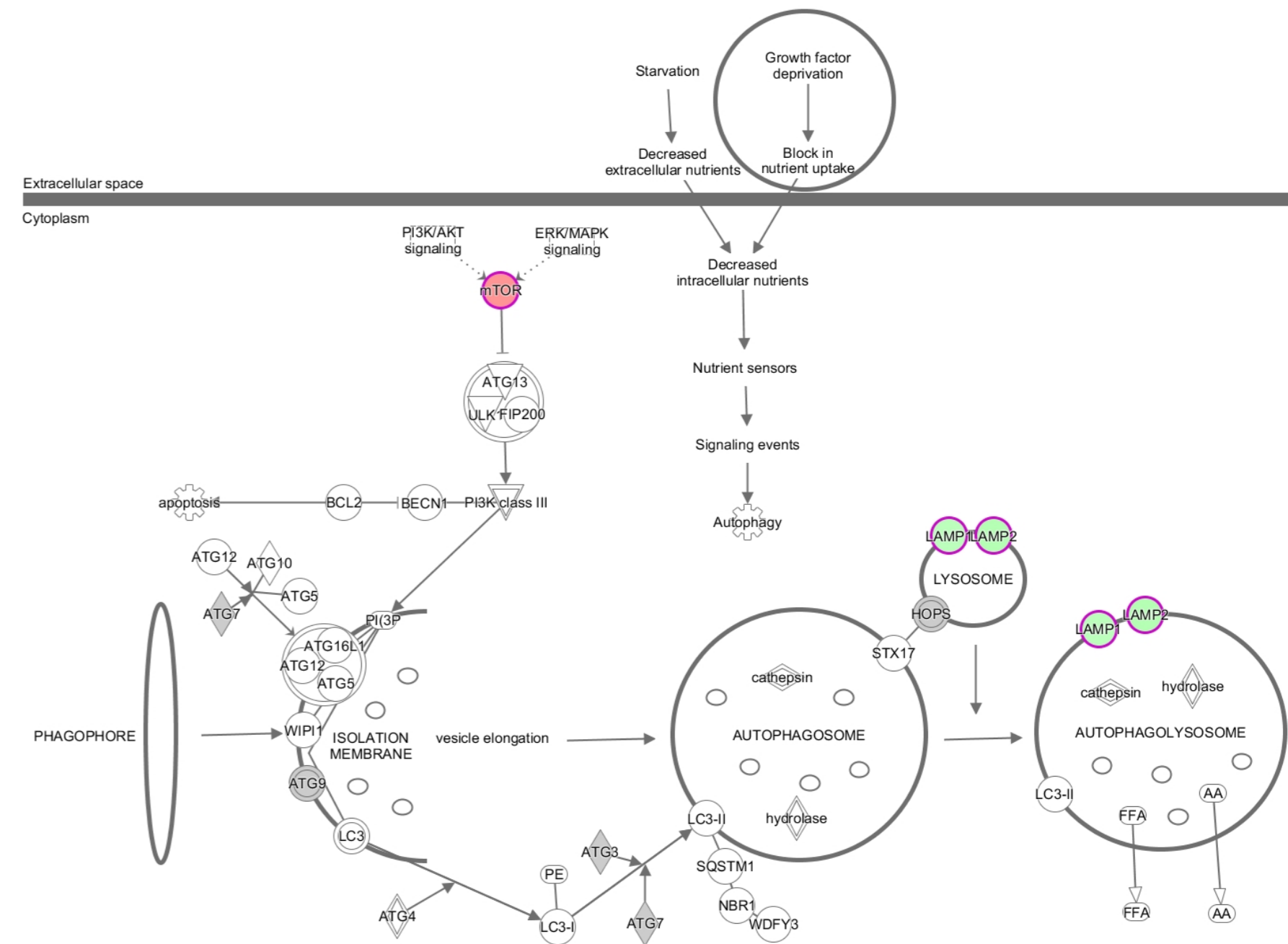

Supplement: Supplementary file 2 — Additional file 2: Figure 32. SILAC-based proteomics identifies cellular response molecules and some related signalling pathways in cells. A Twenty-seven cranial signalling pathways. B Hot-point picture. C BER signalling pathway. D Autophagy signalling. [file 12885_2020_7111_MOESM2_ESM.pdf]
